# Blending citizen science with natural language processing and machine learning: Understanding the experience of living with multiple sclerosis

**DOI:** 10.1371/journal.pdig.0000305

**Published:** 2023-08-02

**Authors:** Christina Haag, Nina Steinemann, Deborah Chiavi, Christian P. Kamm, Chloé Sieber, Zina-Mary Manjaly, Gábor Horváth, Vladeta Ajdacic-Gross, Milo Alan Puhan, Viktor von Wyl

**Affiliations:** 1 Institute for Implementation Science in Health Care, University of Zurich, Switzerland; 2 Epidemiology, Biostatistics and Prevention Institute, University of Zurich, Switzerland; 3 Neurocentre, Lucerne Cantonal Hospital, Lucerne, Switzerland; 4 Department of Neurology, Inselspital, Bern University Hospital, University of Bern, Switzerland; 5 Department of Neurology, Schulthess Klinik, Zurich, Switzerland; 6 Department of Health Sciences and Technology, ETH Zurich, Zurich, Switzerland; Mayo Clinic Arizona, UNITED STATES

## Abstract

The emergence of new digital technologies has enabled a new way of doing research, including active collaboration with the public (‘citizen science’). Innovation in machine learning (ML) and natural language processing (NLP) has made automatic analysis of large-scale text data accessible to study individual perspectives in a convenient and efficient fashion. Here we blend citizen science with innovation in NLP and ML to examine (1) which categories of life events persons with multiple sclerosis (MS) perceived as central for their MS; and (2) associated emotions. We subsequently relate our results to standardized individual-level measures. Participants (n = 1039) took part in the ’My Life with MS’ study of the Swiss MS Registry which involved telling their story through self-selected life events using text descriptions and a semi-structured questionnaire. We performed topic modeling (‘latent Dirichlet allocation’) to identify high-level topics underlying the text descriptions. Using a pre-trained language model, we performed a fine-grained emotion analysis of the text descriptions. A topic modeling analysis of totally 4293 descriptions revealed eight underlying topics. Five topics are common in clinical research: ‘diagnosis’, ‘medication/treatment’, ‘relapse/child’, ‘rehabilitation/wheelchair’, and ‘injection/symptoms’. However, three topics, ‘work’, ‘birth/health’, and ‘partnership/MS’ represent domains that are of great relevance for participants but are generally understudied in MS research. While emotions were predominantly negative (sadness, anxiety), emotions linked to the topics ‘birth/health’ and ‘partnership/MS’ was also positive (joy). Designed in close collaboration with persons with MS, the ‘My Life with MS’ project explores the experience of living with the chronic disease of MS using NLP and ML. Our study thus contributes to the body of research demonstrating the potential of integrating citizen science with ML-driven NLP methods to explore the experience of living with a chronic condition.

## Author summary

Text data is a powerful way to allow individuals to freely express their experiences in their own words. This is especially powerful when conducting large-scale studies involving and collaborating with the public (‘citizen science’). Historically, text analysis has been time-consuming, but innovations in natural language processing and machine learning, both sub-disciplines of artificial intelligence, have made analysis convenient and efficient. We show how this can be implemented using the example of the Swiss Multiple Sclerosis (MS) Registry, a longitudinal patient-centered project. Persons with MS were asked to tell the story of their life with MS in their own words, guided by open-ended questions. We analyzed which high-level topics of life events participants perceived as central and which emotions were reflected in their text entries. Our findings revealed eight distinct topics: ‘diagnosis’, ‘medication/treatment’, ‘rehabilitation/wheelchair’, ‘injection/symptoms’, ‘relapses/children’, ‘work’, ‘birth/health’, and ‘partnership/MS’. Emotions linked to most text entries was predominantly negative (feelings of sadness, anxiety), with the exception of the topics ’Birth/Health’ and ’Partnership/MS’, where the associated emotions were also positive (feelings of joy). Using the Swiss MS Registry as a flagship project, our research attests to research investigating how innovations in NLP and ML can facilitate en-par collaboration with citizens.

## Introduction

The continuous development of digital technologies creates new avenues for the study of complex health conditions [1,2]. Flexible and intuitive online study environments have facilitated convenient recruitment of large and diverse participant samples, circumventing many typical barriers to physical participation (e.g., geographic distance) [3,4]. This new way of doing research coincides with the advancement of ‘citizen science’ approaches in the health sciences. The term ‘citizen science’ refers to the active engagement and collaboration with the public (i.e., ‘citizens’) in scientific endeavors [5]. This approach may be taken to gain novel and first-hand insight into a particular topic or address scientific and societal challenges [6]. In the health sciences, it also reflects an attempt to describe and understand health and illness from the perspective of affected individuals and to incorporate their preferences (e.g., with respect to research topics), experiences, and knowledge (e.g., in interpretation of findings) into the central focus of a research project [7,8].

Previous citizen science studies in the health domain often relied on interviews and personal interactions to engage with citizens and were thus often limited in size by available financial resources [9,10]. An alternative way of engaging citizen scientists at a potentially larger scale is through written free-text and survey-based methodologies [11]. This format allows individuals to express their unique perspective and experiences in their own words and with as much detail as they want, thus allowing insight into content and meaning that often is missed in standardized measurements. Further, recent advances in natural language processing (NLP; e.g., pretrained language models and open text resources) have made the automated analysis of text fast and much more accessible [12]. Large-scale practice applications of NLP involving citizens include, for example, the identification of topics from word clusters that are embedded in large amounts of text [13]. The innovative aspects in recent NLP research were enabled by advances in computing power and open-source machine learning (ML) developments, including, for instance, neural networks that are trained on large-scale text sources (‘language models’) [14]. These types of language models can be utilized for a broad range of complex tasks, including but not limited to, (offline) automated text translation or emotion detection. Emotion analysis quantifies the degree to which text contains indicators of coarse-grained (positive, negative) or fine-grained emotionality (e.g., joy, sadness) [15]. Text-based indicators of emotions have been linked to measures of mental and physical health as well as personality variables [16]. A recent study found that in a community-recruited sample more extensive active and emotionally positive vocabulary was linked to higher wellbeing and better physical health, while a larger active and emotionally negative vocabulary showed the opposite effect [17]. These NLP innovations make free text a rich and convenient source for deep insight into unique individual experiences and perspectives.

The citizen science approach and novel NLP methods lend themselves particularly well to the study of multiple sclerosis (MS) [11]. MS is a severe, prevalent, and chronic neurodegenerative health condition characterized by extensive and highly variable physical and mental symptomatology [18]. The unpredictable and often idiomatic nature of MS is, in and of itself, a substantial source of distress for persons with MS [19]. MS is an ideal use case for ‘citizen science’ because MS is chronic and very heterogeneous, and persons with MS are often very young when diagnosed, leading to a variety of different needs [20,21]. MS is a highly heterogeneous disease in terms of symptoms and progression, and there is no proven cure for MS. As a result, disease management needs to be highly personalized, taking into account many aspects of daily life and lifestyle. Persons with MS often want to be involved in management decisions, in our experience more so than with many other chronic diseases. MS care has a long tradition of patient involvement and shared decision-making between patients and physicians. Our experience with the SMSR suggests that, as a result, persons with MS are often highly motivated to participate in research—both as data contributors and as citizen scientists.

Research aimed at capturing the lived experience of living with MS therefore needs to account for the very diverse experiences and avoid overly restrictive survey designs that would hinder the capture of this heterogeneity. Involving people with MS as citizen scientists at an early stage of the project helps to plan the project and survey in an exploratory manner from the outset, so that we can uncover new facets of the experience of living with MS that were not anticipated in advance.

While the symptomatology of MS has been well studied, comparatively little is known about how persons with MS perceive their health and how they live with MS over longer time periods. Everyday coping with symptom management, relapses, and the general unpredictability of MS symptomatology, often represent overwhelming challenges for the individual that impact significantly on their quality of life and mental health [22,23,24]. Being diagnosed with MS is a turning point for most people and may compel them to reassess not only their life goals, but also who they are as individuals. Added to this identity transformation, they also face the growing physical and mental burden of their disease in their everyday lives [25]. Previous research has found that receiving the initial MS diagnosis may trigger reactions of denial, reduced confidence, and/or concealment [26]. Reconciling one’s identity with the diagnosis seems to be key in terms of the individual’s psychosocial adaptation to their new situation. Some persons with MS report that their personal connections with others become stronger over time and that they reprioritize what is important to them in life [27,28].

Here, we present the citizen science project ‘My life with MS’ conducted by the Swiss MS Registry (SMSR), developed in close cooperation and through the initiative of persons with MS. The lack of large-scale investigations of the first-hand *lived experience* of MS was the basis for the citizen science project ‘My life with MS’. The project’s main purpose was to examine how persons with MS experience living with the disease by blending large-scale citizen science with open-source NLP and ML methods. We examined (1) which general categories of life events persons with MS perceived as central in terms of the nature and progression of their MS across their life course; (2) how those life events resonated emotionally in hindsight; and (3) how the experience of specific MS-related events relates to individual-level characteristics. We expected that participants would describe ‘typical’ MS-related turning points that would relate to interactions with medical practitioners (such as diagnosis, medication, treatment). We further anticipated that they would also mention personal events that resulted from or were shaped by their MS (such as changes to the work situation or the inability to pursue certain hobbies because of MS).

## Methods

### Swiss MS Registry (SMSR)–a longitudinal Citizen Science study

The SMSR is an ongoing longitudinal patient-centered study in Switzerland funded by the Swiss MS Society (https://clinicaltrials.gov/ct2/show/NCT02980640) [11,29]. The study has been approved by the Ethics Committee of the Canton Zurich (PB-2016-00894, BASEC2019-01027). All SMSR participants have provided written informed consent. The SMSR pursues the overarching goal of giving a voice to persons with MS. The SMSR actively collaborates with its participants on various projects as the persons with MS themselves are the best experts on their disease. A detailed description of how the SMSR collaborations with its participants is provided in the Supplement (**S1 Text**).

### The ‘My life with MS’ project

The ‘My life with MS’-project has been developed jointly by scientists and persons with MS. The initial project idea has been sparked by the SMSR’s board consisting of approximately 30 persons with MS that continuously advise on the SMSR’s activities. Part of the board’s role is also to bring up themes that are important for persons with MS and not sufficiently covered in the scientific landscape. Given their relevance and the deficient state of research, the board’s members expressed the wish to research individual-level stories of MS in depth from a lifetime perspective. The SMSR subsequently developed the ‘My life with MS’-project in a multistage, iterative process. The project conceptualization phase included two workshops with persons with MS who contributed to project aims and design, reviewed and edited assessment materials, and provided general feedback. The experiences of the board members and the SMSR’s scientific expertise ultimately developed into the ‘My life with MS’-project which invites participants to tell the story of their life with MS focusing on self-chosen key events. Data collection started in July 2019 and is still ongoing.

In the ‘My Life with MS’ survey, participants were asked to tell the story of their life with MS by identifying key MS-related life events that they considered central to the progression of their MS. Participation in SMSR studies is possible both online and by paper-pencil. Online participants of the SMSR had the option to document up to nine such MS-related events. Paper-pencil participants received sheets for up to three events but could request more sheets if needed. The ‘My life with MS’-survey started with a visual timeline covering the time period between receiving the MS diagnosis and the present day, participants filled in keywords at appropriate points on the timeline, detailing life events/experiences they would like to elaborate on in detail later. For each of these life events, participants completed a brief semi-structured survey. An overview of the survey structure for the individual MS-related events can be found in the Supplement (**S1 Fig**). Participants first provided a short text description of the event itself, the event’s consequences for them personally, sources of support which they had access to at the time, what they felt that they could have used at the time to deal with the event, and their advice for other persons with MS in similar situations. They then allocated the life events to a range of thematic categories, defined as ‘personal level’ (e.g., mental wellbeing), ‘societal level’ (e.g., social position), and ‘direct environment’ (e.g., family and loved ones). They then indicated on a visual analogue scale and as a numeric value how they felt when the event took place (items adapted from the EQ-5D; range: 0–100, higher values reflecting a better emotional state). [30] In addition, participants further specified their emotional state by indicating which negative or positive emotions (happy, relaxed, sad, depressed, anxious, nervous, tired, other) best reflected their emotional state at that time. Next, they provided a free text description of the life event’s impact on their lives. On a 5-point Likert scale, they further rated the perceived impact of the event in hindsight (very negative, negative, neutral, positive, very positive) as well as the persistence of any event-related consequences (brief, recurrent, permanent [always present], other). They subsequently indicated whether the event had any impact on their broader social network (i.e., family, loved ones) and again, rated the event in terms of impact and persistence. Participants then described in free text who had supported them during the event and, in another text entry, what they felt could have helped them in the given situation. Finally, participants were asked to share their advice for other persons in a similar situation. For most of the closed questions, participants had the opportunity to provide additional comments if they wished. The first life event description was followed by self-report questionnaires covering information on participants’ MS disease, medication, therapies, as well as general physical and mental health over the course of the last half year. Participants then completed the procedure described above for the remaining life events.

### Description of the text corpus underlying this study

For the present study, we focused on the event keywords provided in the visual timeline (**S1 Fig**, survey section A1) and the related event descriptions (**S1 Fig**, survey section A3). We combined the keywords with the respective event description for all subsequent analyses. That is because not all participants provided a text description complementing their keywords so that these events would otherwise be lost. Again others, regarded the event description as a continuation of the event keywords so that they did not reiterate the keyword in their description.

### Additional measures

For comparison with the emotion classifications derived from the language model, we used the corresponding survey ratings presented in **S1 Fig** (survey section A7).

### Analytic approach

**Fig 1:**
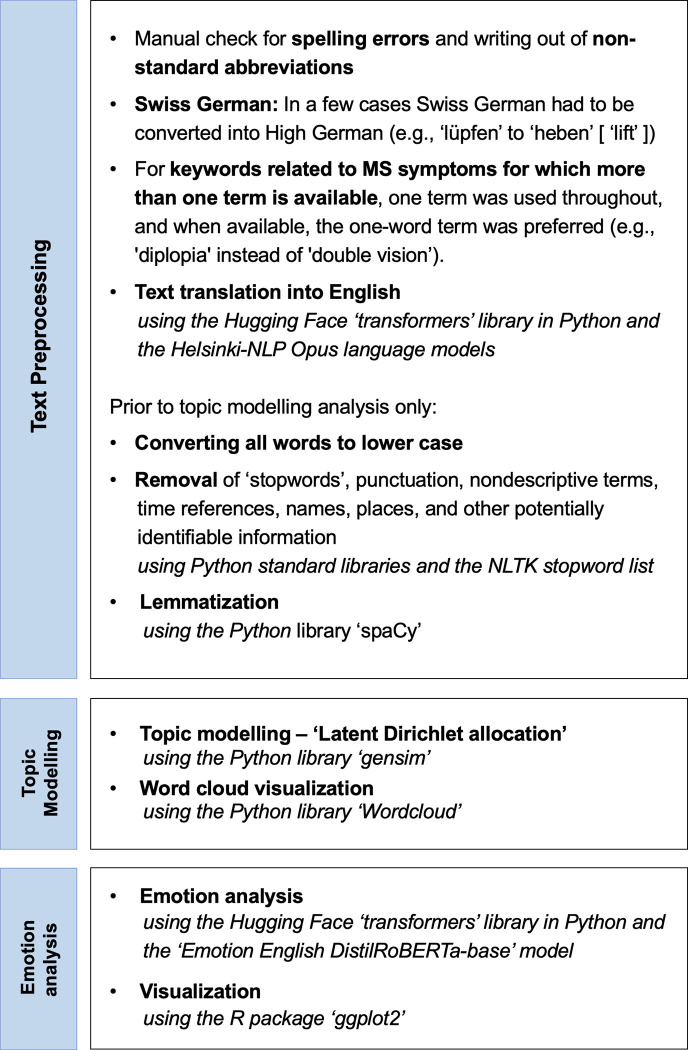
Flowchart visualizing the analytical approach and individual steps in the present manuscript.

The present research implements a range of different analysis methods. To determine which broader categories of life events persons with MS perceived as central and emotions associated with the descriptions, we implemented NLP techniques including topic modelling. To compare variables across groups, we used descriptive statistical methods. All analysis steps are detailed in the following sections. A flowchart visualizing the individual analysis steps is provided in **Fig 1**.

### ML-driven natural language processing

#### Software

All NLP analyses were conducted in Python, version 3.7. using the PyCharm environment, version 2021.3.2. Individual analysis steps and specific Python libraries are detailed in the sections below. All visualizations were completed in R (version 4.1.0), in an RStudio environment (version 1.4.1717) with respective packages detailed below.

### Text preprocessing

Text preprocessing prior to the analyses comprised manual spelling correction, automated language detection, and offline-translation of all the text entries to English, conversion of, for example, clinic names and medication brands into generic terms, removal of stop words and transformation of generic terms, lemmatization, and part-of-speech tagging (explained in more detail below).

All text entries were first checked manually for spelling errors and any abbreviations were spelled unless they were widely established (such as ‘MS’ for ‘multiple sclerosis’ or ‘MRI’ for ‘Magnet Resonance Imaging’). Filling in questionnaires can be exhausting for some persons with MS. Apart from typical spelling errors, our text data is thus characterized by the frequent usage of non-standard abbreviations (e.g., ‘Betaf.’ for ‘Betaferon’). Given the specific nature of errors sources and the variety of MS-specific expressions in our data, we initially conducted a manual spelling correction which also spelled out all uncommon abbreviations. On very rare occasions, participants also used Swiss-German words (e.g., ‘lüpfen’ instead of ‘heben’ [‘lift’]). In such cases, Swiss German words were replaced with High German. Since the SMSR conducts its assessments in the three of the Swiss official languages (i.e., German, French, Italian), we then translated all text entries into English. For keywords related to MS symptomatology for which more than one term is available, one term was consistently used, and if available, the one-word term was preferred (e.g., ‘diplopia’ instead of ‘double images’). For text translation, we used the Hugging Face ‘transformers’ library in Python and the Helsinki-NLP Opus language models ‘German-English’, ‘French-English’, and ‘Italian-English’ which can be retrieved from the Hugging Face website (https://huggingface.co/) [15]. All language models were downloaded, and all translations were completed locally in an offline Python environment. We then automatically removed any names (personal, institutions) and places (cities, countries) from the data for anonymization purposes. A subsequent manual check ensured the data’s anonymization. In this step we additionally excluded, e.g., highly specific job descriptions. We subsequently removed any words classified as ‘stopwords’ (that is, common words without specific meaning such as ‘and’) using the ‘stopword’ list of the widely established ‘NLTK’ library [31]. Given the retrospective nature of the study, we expanded the stopword list by a set of nondescriptive terms and time references which repetitively occurred in individuals’ descriptions (e.g., ‘year’, ‘month’, ‘week’, ‘minute’ etc.). We then lemmatized all words using NLTK’s ‘WordNetLemmatizer’. Lemmatization refers to a word’s conversion to its inflective form (e.g., ‘treatments’ to ‘treatment’) so that different derivations of the same word can be analyzed as a unit. As nouns were most informative for our guiding research aim (i.e., which broader categories of life events persons with MS perceived as crucial in terms of the progression of their MS across the life course), we finally extracted all lemmatized nouns from the text entries using the part-of-tagging functionality implemented in the ‘spacy’ library, version 3.2.3 [32]. Finally, we conducted a manual check of the lemmatized nouns to ensure that only anonymous information was included and that only nouns which can found be in English dictionaries were included. Given the novelty of our methods and the specific nature of our data, we manually checked all preprocessing steps and made corrections where needed.

### Topic modelling—Latent Dirichlet Allocation (LDA)

To identify different types of latent life-event topics, we implemented topic modelling. Topic modelling is an unsupervised ML method suitable to analyze large-scale unlabeled text data based on word co-occurrences. The overarching idea of the method is that a distinct number of latent topics underlies the text entries and, in parallel, each of the text entries is a composite of different topics in varying proportions. LDA is an established probabilistic modelling technique [33]. The process of topic determination through LDA works such that words are allocated to a topic on the basis of their co-occurrence with other words in the text entries. For this study, we used the LDA algorithm implemented in the ‘gensim’ library, version 3.8.3 [34].

*Rationale for topic determination*. A challenge and controversial issue in topic modelling is the determination of the optimal number of topics to be modeled. This choice is key as it has to be decided on prior to the LDA analysis and thereby impacts on the nature of the topics to be generated [35,36,37]. Following recent recommendations and subsequent applications, we combined a data-driven and empirical approach to determine the optimal number of topics [38,39]. Specifically, we generated a series of models for comparison purposes. We started with a two-topics model and subsequently increased number of topics by one until 40 (that is, a 40-topics model). We checked different topic models manually for coherence and interpretability by exploring how the topic representations changed in terms of their keywords and the keywords’ importance as we successively increased the number of topics. To ensure that our interpretation of the respective topic representations was correct, we continuously verified our understanding with the underlying text data that was representative of the respective topics (i.e., which had a high probability score in the LDA model). Increasing the number of topics generally results in splitting topics that were previously one into smaller subtopics. A key challenge in this process is to strike a balance between capturing the larger underlying patterns of the corpus without neglecting important details. Finally, we compared the models’ goodness in terms of their topic’s coherence (i.e., the ‘c_v score’), which provides a statistical proxy in this respect. While the ‘c_v score’ can be considered as a general reference point, domain expert knowledge is key for fine-tuning topic models to ensure that topics are coherent and well-interpretable at the content level. In line with Occam’s razor, we opted for an LDA model optimizing topic coherence while being as parsimonious as possible.

*Clear allocation to a distinct category*. We considered an event to be clearly allocated to a certain category (1) if it was the dominant category (that is, the highest percentage of the topic probability score), and (2) if this probability score was larger than 33%. We decided on the one-third allocation upon prior exploration of the probability score distributions in the present sample and found this cut-off to be appropriate for noise reduction purposes.

*Visualization*. To visualize the distinct topics in an information-dense fashion, we computed ‘word clouds’ using the ‘wordcloud’ library, version 1.8.1 [40]. The ‘word clouds’ consist of the most frequent key words underlying a specific topic whereas the word size corresponds to their relative frequency of its occurrence across all text entries.

### Fine-grained emotion analysis

We performed the emotion analysis based on the text entries. While study participants documented a high number of event descriptions (n = 4297), they completed the full event questionnaire, including self-rated emotion categorizations, for only 58.72% of all events (n = 2523). This was most common when an individual documented many events. We conducted the emotion analysis using the full, translated data including in English language in which translation errors had been corrected, all abbreviations were written out in full (e.g., ‘multiple sclerosis’ instead of ‘ms’), specific terms such as medication names had been converted to generic expressions (e.g., ‘medication’ instead of ‘Interferon’) and which still included all stopwords, verbs, adjectives, nouns, and punctuation.

*Rationale for model selection*. We determined the event descriptions’ emotional content using a pretrained language model from the ‘Hugging Face’ open-source platform and community [15]. To decide on a pretrained emotion classification model, which is suitable for our type of text data, we conducted an upfront evaluation of the available models by assessing their classification performance on our data. Our prior evaluation found the ‘Emotion English DistilRoBERTa-base’ model to be most appropriate for the ‘My life with MS’-data [41]. This pretrained language model outputs a probability score for each of the six emotions theorized by Paul Ekman as ‘basic human emotions’ (‘fear’, ‘joy’, ‘sadness’, ‘surprise’, ‘anger’, ‘disgust’) together with a neutral category. The ‘Emotion English DistilRoBERTa-base’ model is based on the distilled version of the original ‘RoBERTa-base model’ which is smaller in size but has similar performance compared to the original. The model is suitable text data in English language. For the emotion classification task, the ‘distilled RoBERTa-base model’ has been fine-tuned on six publicly available datasets including data labelled with the respective emotions [42].

*Analysis*. For emotion determination in Python, we used ‘Transformers’ library (https://huggingface.co/transformers) which has been developed by ‘Hugging Face’ to make the website’s models conveniently accessible to the community [15]. The Transformers library provides application programming interfaces and tools for download and training of open-source pre-trained models that are available on the website. For emotion analysis, we first loaded the model into Python. We then converted the text data into a numeric format for further processing of the text data using the ’RobertaTokenizerFast’ tokenizer. This tokenizer is suitable for any language model based on the ’RoBERTa model’ [43]. Each unique participant text entry was assigned a score for each of the six represented emotions. We then normalized the model output for respective emotions using the ‘softmax’ function from the ‘scipy’ Python library [44]. This transformation allows the results to be interpreted as probabilities which makes it more intuitively understandable [45]. To compare the emotion scores derived from this analysis with the corresponding, binary ratings from the survey, we calculated point-biserial correlations. We then visualized the resulting correlation using the R package ‘corrplot’, version 0.92 [46].

*Visualizations*. Data visualizations were computed using the ‘gglot2’ library, version 3.3.5, in R. We used box plots for a standardized visualization of the eight topics’ fine-grained emotion characteristics with the raw data displayed as dots.

## Results

### Descriptive analyses

All SMSR participants were invited to participate in the present study (n = 2602, status March 2022) and 1039 (39.93%) of them participated in the ‘My life with MS’ survey, either online (n = 873; 84.02%) or as paper-pencil version (n = 166; 15.98%). The average age of participants was 48.55 years (SD = 12.58 years; range: 19–83 years) and 75.65% were female. Participants provided a total of 4309 event descriptions. Sixteen event descriptions had to be excluded because they did not contain meaningful information (e.g., due to the use of unusual abbreviations). This resulted in a final sample of 4293 event descriptions with an average 4.13 events per person (SD: 2.21; range: 1–11; **S2 Fig**). The translated text descriptions (still including all stopwords) were on average of 18.27 words in length (SD: 29.57; range: 1–253). Of the 1039 participants, 79.30% provided their text entries in German (n = 824), 17.61% in French (n = 183), 2.79% in Italian (n = 29), and 0.28% in English (n = 3).

### Central life events for the progression of the MS

**Fig 2:**
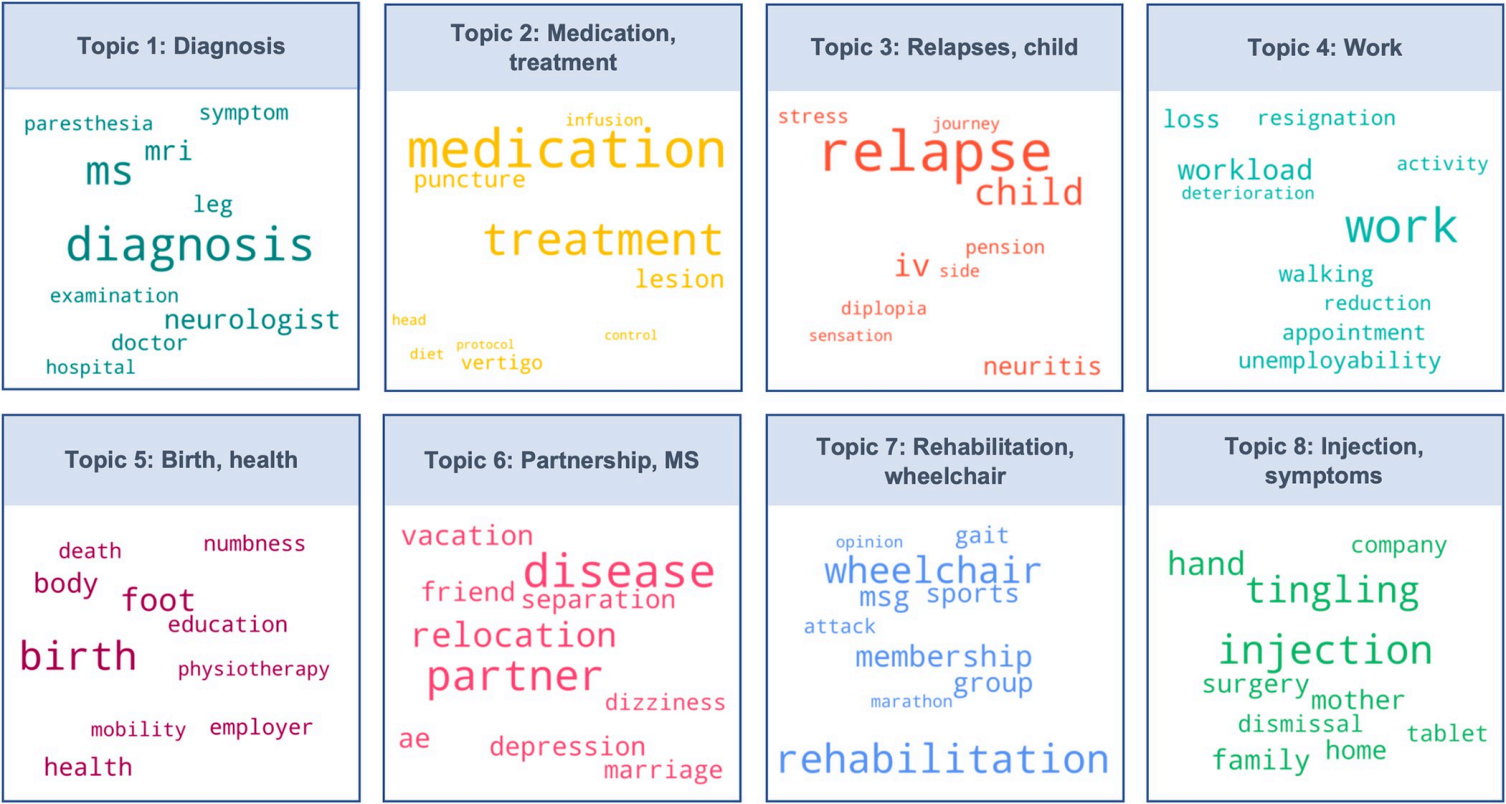
Word cloud visualizing the ten most frequent key words of the 8 overarching topics of MS-related life events, displayed for each of the eight topics separately. Word size corresponds to a specific word’s relative frequency compared to the total number of analyzed words. The words’ color is distinct for the eight topics. AE = adverse effects; IV = abbreviation for the disability insurance in Switzerland; MSG = Swiss MS Society.

First, we examined which broader categories of life events (‘topics’) persons with MS perceived as central in terms of the progression of their MS. Modelling coherence values for an increasing number of topics showed highest coherence value for an LDA models with 8 topics (and with 30 or more topics; **S3 Fig**). In line with Occam’s razor, we opted for an LDA model optimizing topic coherence while being as parsimonious as possible. Among those options, manual examination confirmed that modelling eight topics led to the most distinct topics and thereby best facilitated their interpretation. The eight topics were (1) ‘diagnosis’, (2) ‘medication, treatment’, (3) ‘relapse, child’, (4) ‘work’, (5) ‘birth, health’, (6) ‘partnership, MS’, (7) ‘rehabilitation, wheelchair’, and (8) ‘injection, symptoms’. The 10 most frequent keywords per topic are displayed in **Fig 2** with font size reflecting a words’ relative frequency. Exemplary, anonymized event descriptions which are most representative for each of the topics are provided in **Table 1**.

**Table 1:** Characterization of the eight overarching topics based on the most frequent keywords and exemplary, anonymized event descriptions for the most frequent emotions.

	Topic	Keywords	Emotions	*Exemplary*, *anonymized event descriptions*
**1**	**Diagnosis (n = 827)**	diagnosis, MS, MRI neurologist, leg, doctor, symptom, paresthesia, examination, hospital	**Sadness**	*I received the MS diagnosis*. *I was devastated and very sad*. *I was no longer able to cope with everyday stressful situations*.
			**Fear**	*I could not feel my legs anymore properly*. *Several investigations and an MRI finally revealed I have MS*. *I was deeply frightened*.
			**Joy**	*When I received the diagnosis*, *I was relieved—finally I had an explanation for my unclear symptoms*. *In addition*, *I was no longer considered a fake*.
**2**	**Medication, treatment (n = 539)**	medication, treatment, lesion, puncture, vertigo, infusion, head, control, protocol, accident	**Sadness**	*I had to take medication to fight the MS symptoms*, *but the medication itself was making me feel sick*. *I felt like I was in a vicious cycle and did not know how to get out of it*. *I was devastated*.
			**Fear**	*I am aware that the medications are an interference to the natural functions of my body*. *That scares me*.
			**Joy**	*Starting with the medication gave me confidence that I could remain independent*.
**3**	**Relapses (n = 147)**	relapse, child, IV, neuritis, stress, pension, diplopia, side, journey, sensation	**Sadness**	*Despite taking medication*, *I suffered a severe relapse*. *I could no longer manage my daily tasks and became very depressed*.
			**Fear**	*A tingling sensation spread through my body*, *and I could no longer walk properly*. *I didn’t know what was going on and I was scared*.
			**Joy**	*I had relapses until I got support from IV*. *Since then*, *no more relapses—stress seems to drive the MS*.
**4**	**Work (n = 193)**	work, workload, loss, unemployability, walking, appointment, resignation, reduction, activity, deterioration	**Sadness**	*Despite all possible measures and great support in the team*, *I was unable to continue my work—farewell to an important time in my life*.
			**Fear**	*The increasing symptom burden made it impossible for me to do my job as I used to*. *I was afraid of being fired*.
			**Joy**	*Professional reorientation*. *The reorientation was a relief for me*, *and suddenly new paths and opportunities emerged*.
**5**	**Birth, health (n = 86)**	birth, foot, body, health, education, employer, numbness, death, mobility, physiotherapy	**Sadness**	*Death of my husband*.
			**Fear**	*I am worried that I will have a relapse after pregnancy*.
			**Joy**	*The birth of my grandchild*. *She loves me and has confidence in me*.
**6**	**Partnership, MS (n = 80)**	disease, partner, relocation, vacation, AE (‘adverse effect’), friend, depression, separation, marriage, dizziness	**Sadness**	*Emotional separation from my husband*. *He could not deal with my disease*. *This made me very sad*.
			**Fear**	*My partner and I became emotionally distant from each other*. *I was afraid of losing everything*.
			**Joy**	*I have found a new partner*. *I feel loved*, *respected*, *and valued*.
**7**	**Rehabilitation, wheelchair (n = 73)**	rehabilitation, wheelchair, membership, MSG, group, sports, gait, attack, marathon, opinion	**Sadness**	*I am increasingly dependent on a wheelchair*. *I had to give up my beloved sports*.
			**Fear**	*The increasing spasticity makes driving dangerous*. *I am afraid of causing an accident and having to give back my driving license*.
			**Joy**	*I have returned from an extended stay in rehabilitation*. *It was tough and intense*, *but I benefited both physically and psychologically*.
**8**	**Injections, symptoms (n = 54)**	injection, tingling, hand, family, mother, home, surgery, dismissal, company, tablet	**Sadness**	*The increasing symptom burden (tingling*, *numbness) causes me to withdraw more and more*. *I slip into a depression*.
			**Fear**	*At some point I was afraid of each new injection—there seemed to be hardly any skin left to stick the needle into*.
			**Joy**	*My symptoms have improved after I started the new treatment*.

*Notes*. The term ‘puncture’ in topic 2 (‘Medication, treatment’) relates either to ‘lumbar puncture’ or ‘spinal puncture’. AE = adverse effects; IV = abbreviation for the disability insurance in Switzerland; MSG = Swiss MS society

### Emotions

**Fig 3:**
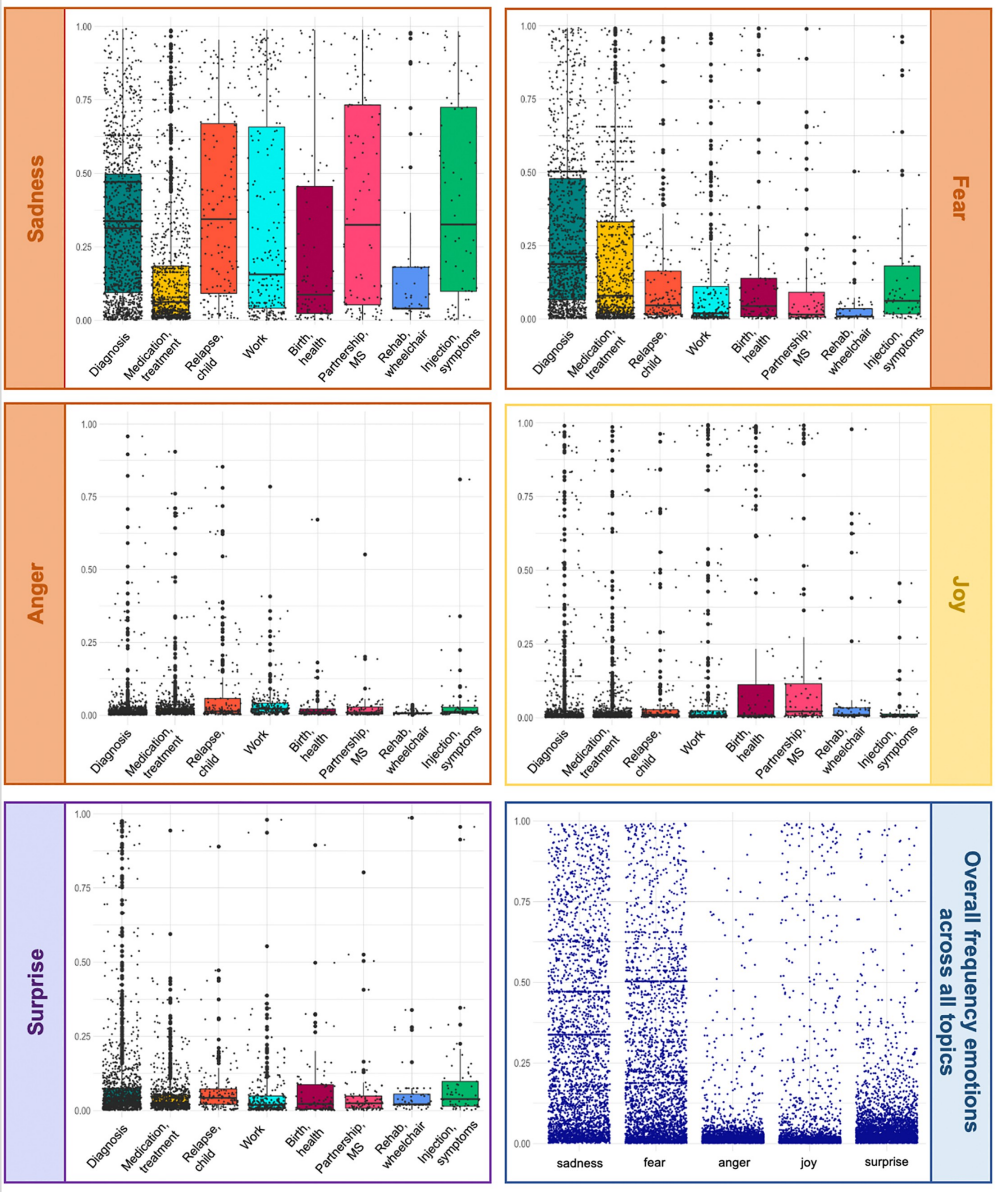
Emotion probability scores for ‘sadness’, ‘fear’, ‘anger’, ‘joy’, ‘surprise’, and the overall frequency of the emotions across all events are displayed separately for the eight topic categories. Only events for which (1) the respective category is the "main category" and (2) the assignment to this main category is at least one third (33%) are included in the graph. Emotion probability scores range from 0 to 1. Higher scores indicate a higher probability for a text description being corresponding to the respective emotion category. Emotion probability scores are displayed using boxplots. The boxplot’s middle line refers to a topic category’s median, the box itself displays the interquartile range (IQR; the range from the 25^th^ to the 75^th^ percentile), and the whiskers mark the minimum (25^th^-1.5*IQR) and maximum (75^th^-1.5*IQR). The thick dots represent outliers beyond the minimum/maximum. The small dots display the raw data.

We subsequently examined the emotions associated with different types of life events (**Fig 3**). We found the key MS-related life events to be predominantly linked to emotions of negative valence, most notably to sadness with means per topic category ranging between 0.41 and 0.15. Fear was also a frequent emotion with means per topic category ranging between 0.21 and 0.04. The emotions ‘anger’, ‘surprise’, and ‘disgust’ were rare among all topic categories. Importantly, feelings of joy were linked to the topics ‘birth / health’ (M = 0.19, SD = 0.34) and ‘partnership & MS’ (M = 0.14, SD = 0.27). It is important to note that the text entries exhibited a high amount of heterogeneity as indicated by the large standard deviations and as visualized in **Fig 3**. A plot which also includes the rare emotion disgust is provided in the supporting information (**S4 Fig**). For 2523 of the 4294 text entries, participants also provided survey ratings (58.76%). We validated the emotion classification based on the pre-trained language model by correlating the respective probability scores of the emotions for each entry with the binary survey ratings (emotion was present vs. emotion was not present at the time) provided by the participants. A correlation plot displaying the point-biserial correlation coefficients for all comparisons is provided in **S5 Fig**. Our results show that the emotions identified by the pre-trained language model are positively correlated with the binary survey ratings (i.e., fear, sadness, joy).

### Topic characterization

The eight topic categories consisted of five directly MS-related topics (‘diagnosis’, ‘medication, treatment’, ‘relapse, child’, ‘injections, symptoms’, ‘rehabilitation, wheelchair’) and three topic categories that address broader life goals (‘partnership, MS’, ‘work, ‘birth, health’). Diagnosis was the most frequent topic category (34.75%), followed by ‘medication, treatment’ (23.64%) and ‘work’ (11.06%). Each of these topic categories was defined by its most frequent terms. In contrast, the topic categories ‘relapse’ (12.30%), ‘birth’ (5.26%), ‘rehabilitation, wheelchair’ (5.33%), or ‘injections, tingling’ (2.84%) were rather heterogeneous in nature. The eight distinct topic categories are detailed below.

### MS-related topics

#### Diagnosis (topic 1)

The 10 most frequent keywords in the first topic category were typical terms related to the MS diagnostic process, e.g., MRI, examinations by neurologists, or common initial symptoms such as paresthesia or visual disturbances. On an emotional level, many individuals expressed feelings of anxiety and sadness in this context, but more positive feelings were also documented, reflecting relief, for example.

### Medication, treatment (topic 2)

The most frequent references in this topic category were related to immunomodulatory treatment, which is usually prescribed to prevent the worsening of a disease. Emotions most frequently associated with this topic category were sadness and fear, possibly related to uncertain treatment outcomes (e.g., continued worsening despite treatment), negative treatment outcomes, or adverse effects.

### Relapses, child (topic 3)

This topic category was mainly defined by the word relapse. However, the additional keywords indicated a rather heterogeneous context of relapse. As the keywords ‘child’ and ‘IV’ (referring to disability insurance) suggest, relapse affects many aspects of the lives of persons with MS. Others may indicate permanent impairments leading to disability (and the need for support from the disability insurance, which in Switzerland is abbreviated to ‘IV’). As a concrete example, some participants expressed fear of relapse during the postpartum period. On the emotional level, text entries are again characterized by sadness and, to a lesser extent, fear.

### Injections, symptoms (topic 8)

This topic category is of a relatively heterogeneous nature. The common feature of the text entries in this category was the constant interference of symptoms (often tingling) with daily life, and the price of controlling them is often high. Reference is made to injections, the usual mode of administration of older disease-modifying drugs. Such injections are painful, and participants report increasing painful indurations under the skin, some of which require surgical removal. Both symptoms and therapy affect all aspects of life.

### ‘Rehabilitation, wheelchair’ (topic 7)

Progressive disability, as indicated by the keyword "wheelchair," for example, was the focus of this topic category. Progressive disability was also associated with the increasing need to rely on walking aids or to cope with more severe impairments (e.g., through rehabilitation measures). Membership of the Swiss MS Society for information and networking purposes was also mentioned in this topic category. Negative emotions were less common in this category, possibly related to the heterogeneous nature of this category. Indeed, rehabilitation was most often perceived as positive, while the need for a wheelchair or the prospect of needing one was perceived as negative.

### Topics concerning broader life goals

#### Partnership, MS (topic 6)

This topic category brings together text entries that relate to both the difficult and positive aspects of partnerships, especially in the context of MS. The common term "separation" refers to breakups, which are often also related to the pressures that MS puts on a partnership. This category of themes was associated with feelings of sadness that may reflect the stress and loss that can be associated with such separations. On the positive side of partnerships, study participants also reported meeting a new partner or feeling a connection with another person. This may be reflected in the feelings of joy associated with this category of issues.

### Work (topic 4)

The work-related consequences of MS are at the core of topic category 4. As terms such as resignation (in the sense of quitting), unemployment, or reduction reflect, this theme category likely reflected the personal struggle that persons with MS experience in remaining employed and managing their daily workload despite multiple impairments. The text entries in this theme category were often characterized by feelings of sadness.

### Birth, health (topic 5)

This topic category was primarily characterized by the term ‘birth’, which mostly refers to the birth of one’s own child or a grandchild. These events were mostly associated with joyful, positive feelings. However, several other prominent keywords indicate considerable heterogeneity in the statements (examples provided in **Table 1**). Other text entries in this topic category tended to focus on education (e.g., continuing education) or symptom management (physical therapy).

### Descriptive characterization of individuals per topic category

A descriptive characterization of study participants based on the topic categories is provided in **Table 2**. In the table, participants are divided into those who made at least one entry on a particular topic category and those who made no such entry. Since the topic categories ‘diagnosis’ and ‘medication / treatment’ were reported by most participants (see **S6 Fig**), we examined only the topics ‘relapse / child’, ‘work’, ‘birth, health’, ‘partnership & MS’, ‘rehabilitation / wheelchair’, and ‘injection, symptoms’. The descriptive analysis revealed, however, that individuals-level characteristics (i.e., MS type, living situation, parentship, etc.) were distributed relatively homogeneous.

**Table 2:** Relationship between six of eight overarching event topics and individual-level measures.

Socio-demographic characteristics	Relapse		Work		Birth, health		Partnership, MS		Rehabilitation, wheelchair		Injections, symptoms	
	Repor- ted (N=147)	N/A (N=843)	Repor- ted (N=193)	N/A (N=797)	Repor- ted (N=85)	N/A (N=905)	Repor- ted (N=80)	N/A (N=910)	Repor- ted (N=73)	N/A (N=917)	Repor- ted (N=54)	N/A (N=936)
**Age**												
Mean (SD)	49.0 (12.0)	48.5 (12.7)	51.3 (11.8)	47.9 (12.7)	50.8 (12.6)	48.3 (12.6)	50.6 (13.9)	48.4 (12.4)	53.5 (12.8)	48.1 (12.5)	50.9 (10.6)	48.4 (12.7)
Median [Min, Max]	50.0 [25.0, 82.0]	49.0 [19.0, 83.0]	52.0 [25.0, 83.0]	48.0 [19.0, 83.0]	52.0 [25.0, 83.0]	49.0 [19.0, 83.0]	52.5 [25.0, 82.0]	49.0 [19.0, 83.0]	56.0 [19.0, 80.0]	49.0 [20.0, 83.0]	49.5 [29.0, 77.0]	49.0 [19.0, 83.0]
**Gender**												
female	116 (78.9%)	640 (75.9%)	142 (73.6%)	614 (77.0%)	68 (80.0%)	688 (76.0%)	64 (80.0%)	692 (76.0%)	53 (72.6%)	703 (76.7%)	41 (75.9%)	715 (76.4%)
male	31 (21.1%)	203 (24.1%)	51 (26.4%)	183 (23.0%)	17 (20.0%)	217 (24.0%)	16 (20.0%)	218 (24.0%)	20 (27.4%)	214 (23.3%)	13 (24.1%)	221 (23.6%)
**MS type**												
Primary-progressive MS (n [%])	13 (8.8%)	96 (11.4%)	24 (12.4%)	85 (10.7%)	18 (21.2%)	91 (10.1%)	9 (11.3%)	100 (11.0%)	10 (13.7%)	99 (10.8%)	5 (9.3%)	104 (11.1%)
Relapsing-remitting MS (n [%])	91 (61.9%)	577 (68.4%)	112 (58.0%)	556 (69.8%)	43 (50.6%)	625 (69.1%)	42 (52.5%)	626 (68.8%)	44 (60.3%)	624 (68.0%)	37 (68.5%)	631 (67.4%)
Secondary-progressive MS (n [%])	25 (17.0%)	105 (12.5%)	38 (19.7%)	92 (11.5%)	15 (17.6%)	115 (12.7%)	14 (17.5%)	116 (12.7%)	16 (21.9%)	114 (12.4%)	10 (18.5%)	120 (12.8%)
Transition (n [%])	8 (5.4%)	31 (3.7%)	10 (5.2%)	29 (3.6%)	6 (7.1%)	33 (3.6%)	5 (6.3%)	34 (3.7%)	3 (4.1%)	36 (3.9%)	2 (3.7%)	37 (4.0%)
CIS (n [%])	3 (2.0%)	20 (2.4%)	4 (2.1%)	19 (2.4%)	1 (1.2%)	22 (2.4%)	7 (8.8%)	16 (1.8%)	0 (0%)	23 (2.5%)	0 (0%)	23 (2.5%)
Missing (n [%])	7 (4.8%)	14 (1.7%)	5 (2.6%)	16 (2.0%)	2 (2.4%)	19 (2.1%)	3 (3.8%)	18 (2.0%)	0 (0%)	21 (2.3%)	0 (0%)	21 (2.2%)
**Marital status**												
Married (n [%])	72 (49.0%)	417 (49.5%)	103 (53.4%)	386 (48.4%)	34 (40.0%)	455 (50.3%)	39 (48.8%)	450 (49.5%)	44 (60.3%)	445 (48.5%)	31 (57.4%)	458 (48.9%)
Divorced (n [%])	16 (10.9%)	87 (10.3%)	23 (11.9%)	80 (10.0%)	15 (17.6%)	88 (9.7%)	10 (12.5%)	93 (10.2%)	6 (8.2%)	97 (10.6%)	2 (3.7%)	101 (10.8%)
Registered partnership (n [%])	2 (1.4%)	12 (1.4%)	4 (2.1%)	10 (1.3%)	0 (0%)	14 (1.5%)	4 (5.0%)	10 (1.1%)	1 (1.4%)	13 (1.4%)	0 (0%)	14 (1.5%)
Separated (n [%])	1 (0.7%)	13 (1.5%)	0 (0%)	14 (1.8%)	2 (2.4%)	12 (1.3%)	3 (3.8%)	11 (1.2%)	2 (2.7%)	12 (1.3%)	0 (0%)	14 (1.5%)
Unmarried (n [%])	40 (27.2%)	246 (29.2%)	49 (25.4%)	237 (29.7%)	25 (29.4%)	261 (28.8%)	21 (26.3%)	265 (29.1%)	13 (17.8%)	273 (29.8%)	19 (35.2%)	267 (28.5%)
Widowed (n [%])	2 (1.4%)	16 (1.9%)	1 (0.5%)	17 (2.1%)	1 (1.2%)	17 (1.9%)	2 (2.5%)	16 (1.8%)	3 (4.1%)	15 (1.6%)	2 (3.7%)	16 (1.7%)
Other (n [%])	0 (0%)	3 (0.4%)	1 (0.5%)	2 (0.3%)	0 (0%)	3 (0.3%)	0 (0%)	3 (0.3%)	0 (0%)	3 (0.3%)	0 (0%)	3 (0.3%)
Missing (n [%])	14 (9.5%)	49 (5.8%)	12 (6.2%)	51 (6.4%)	8 (9.4%)	55 (6.1%)	1 (1.3%)	62 (6.8%)	4 (5.5%)	59 (6.4%)	0 (0%)	63 (6.7%)
**Living situation**												
With family (n [%])	37 (25.2%)	258 (30.6%)	51 (26.4%)	244 (30.6%)	23 (27.1%)	272 (30.1%)	16 (20.0%)	279 (30.7%)	21 (28.8%)	274 (29.9%)	17 (31.5%)	278 (29.7%)
with friends / relatives / in shared flat (n [%])	2 (1.4%)	15 (1.8%)	3 (1.6%)	14 (1.8%)	1 (1.2%)	16 (1.8%)	3 (3.8%)	14 (1.5%)	0 (0%)	17 (1.9%)	0 (0%)	17 (1.8%)
alone / single-parenting (n [%])	25 (17.0%)	181 (21.5%)	37 (19.2%)	169 (21.2%)	19 (22.4%)	187 (20.7%)	19 (23.8%)	187 (20.5%)	15 (20.5%)	191 (20.8%)	14 (25.9%)	192 (20.5%)
Parents (n [%])	1 (0.7%)	20 (2.4%)	5 (2.6%)	16 (2.0%)	4 (4.7%)	17 (1.9%)	2 (2.5%)	19 (2.1%)	1 (1.4%)	20 (2.2%)	1 (1.9%)	20 (2.1%)
With spouse/ partner (n [%])	69 (46.9%)	316 (37.5%)	85 (44.0%)	300 (37.6%)	29 (34.1%)	356 (39.3%)	37 (46.3%)	348 (38.2%)	32 (43.8%)	353 (38.5%)	21 (38.9%)	364 (38.9%)
At clinic /residential home /therapeutic residential community (n [%])	1 (0.7%)	5 (0.6%)	1 (0.5%)	5 (0.6%)	1 (1.2%)	5 (0.6%)	2 (2.5%)	4 (0.4%)	0 (0%)	6 (0.7%)	1 (1.9%)	5 (0.5%)
Other (n [%])	0 (0%)	2 (0.2%)	1 (0.5%)	1 (0.1%)	0 (0%)	2 (0.2%)	0 (0%)	2 (0.2%)	0 (0%)	2 (0.2%)	0 (0%)	2 (0.2%)
Missing (n [%])	12 (8.2%)	46 (5.5%)	10 (5.2%)	48 (6.0%)	8 (9.4%)	50 (5.5%)	1 (1.3%)	57 (6.3%)	4 (5.5%)	54 (5.9%)	0 (0%)	58 (6.2%)
**Parentship**												
Yes (n [%])	69 (46.9%)	444 (52.7%)	96 (49.7%)	417 (52.3%)	44 (51.8%)	469 (51.8%)	37 (46.3%)	476 (52.3%)	44 (60.3%)	469 (51.1%)	28 (51.9%)	485 (51.8%)
No (n [%])	60 (40.8%)	298 (35.4%)	75 (38.9%)	283 (35.5%)	30 (35.3%)	328 (36.2%)	33 (41.3%)	325 (35.7%)	20 (27.4%)	338 (36.9%)	26 (48.1%)	332 (35.5%)
Missing (n [%])	18 (12.2%)	101 (12.0%)	22 (11.4%)	97 (12.2%)	11 (12.9%)	108 (11.9%)	10 (12.5%)	109 (12.0%)	9 (12.3%)	110 (12.0%)	0 (0%)	119 (12.7%)
**Work**												
(Self-)employed (n [%])	82 (55.8%)	514 (61.0%)	119 (61.7%)	477 (59.8%)	51 (60.0%)	545 (60.2%)	35 (43.8%)	322 (35.4%)	31 (42.5%)	565 (61.6%)	34 (63.0%)	562 (60.0%)
Unemployed (n [%])	58 (39.5%)	299 (35.5%)	71 (36.8%)	286 (35.9%)	31 (36.5%)	326 (36.0%)	45 (56.3%)	551 (60.5%)	41 (56.2%)	316 (34.5%)	20 (37.0%)	337 (36.0%)
Missing (n [%])	7 (4.8%)	30 (3.6%)	3 (1.6%)	34 (4.3%)	3 (3.5%)	34 (3.8%)	0 (0%)	37 (4.1%)	1 (1.4%)	36 (3.9%)	0 (0%)	37 (4.0%)
**Self-reported disability (SRDSS)**												
EDSS 0-3.5 (no walking aids; n [%])	89 (60.5%)	586 (69.5%)	114 (59.1%)	561 (70.4%)	45 (52.9%)	630 (69.6%)	50 (62.5%)	625 (68.7%)	41 (56.2%)	634 (69.1%)	38 (70.4%)	637 (68.1%)
EDSS 4-6.5 (use of walking aids; n [%])	40 (27.2%)	164 (19.5%)	51 (26.4%)	153 (19.2%)	27 (31.8%)	177 (19.6%)	15 (18.8%)	189 (20.8%)	19 (26.0%)	185 (20.2%)	12 (22.2%)	192 (20.5%)
EDSS 7-10 (use of wheelchair; n [%])	11 (7.5%)	59 (7.0%)	23 (11.9%)	47 (5.9%)	10 (11.8%)	60 (6.6%)	15 (18.8%)	55 (6.0%)	10 (13.7%)	60 (6.5%)	4 (7.4%)	66 (7.1%)
Missing (n [%])	7 (4.8%)	34 (4.0%)	5 (2.6%)	36 (4.5%)	3 (3.5%)	38 (4.2%)	0 (0%)	41 (4.5%)	3 (4.1%)	38 (4.1%)	0 (0%)	41 (4.4%)
**Health-related quality of life (EQ-5D)**												
EQ-5D sum score (mean [SD])	10.1 (3.40)	9.14 (3.74)	10.2 (3.63)	9.04 (3.69)	10.3 (3.72)	9.18 (3.69)	10.2 (3.87)	9.20 (3.68)	10.2 (3.77)	9.20 (3.69)	9.72 (3.33)	9.26 (3.73)
Missing (n [%])	23 (15.6%)	145 (17.2%)	24 (12.4%)	144 (18.1%)	7 (8.2%)	161 (17.8%)	10 (12.5%)	158 (17.4%)	6 (8.2%)	162 (17.7%)	7 (13.0%)	161 (17.2%)
EQ-5D VAS (mean [SD])	68.1 (23.2)	71.6 (21.8)	66.3 (22.5)	72.3 (21.8)	66.8 (25.2)	71.5 (21.7)	68.1 (22.9)	71.3 (22.0)	72.3 (19.5)	70.9 (22.3)	68.6 (20.5)	71.2 (22.1)
Missing (n [%])	24 (16.3%)	139 (16.5%)	22 (11.4%)	141 (17.7%)	6 (7.1%)	157 (17.3%)	10 (12.5%)	153 (16.8%)	5 (6.8%)	158 (17.2%)	7 (13.0%)	156 (16.7%)

*Notes*. The ‘reported’ column includes the number of study participants which have reported at least one event that can clearly be allocated to the respective topic categories. ‘N/A’ refers to participants who did not report an event that could be assigned to a given topic category.

## Discussion

Designed in close collaboration with persons with MS, the ‘My Life with MS’ project explores the experience of living with the chronic disease of MS using recent innovations in NLP and ML. Our study thus contributes to the body of research demonstrating the potential of integrating citizen science with ML-driven NLP methods to explore the experience of living with a chronic condition. Over 1000 persons with MS shared their experiences in the semi-structured survey, ‘My life with MS’. A topic modelling analysis revealed that key MS-related life events clustered into eight overarching categories: (1) ‘diagnosis’, (2) ‘medication/treatment’, (3) ‘relapses/children’, (4) ‘work’, (5) ‘birth/health’, (6) ‘partnership/MS’, (7) ‘rehabilitation/wheelchair’, and (8) ‘injection/symptoms’. Emotions associated with the text entries was predominantly negative, with sadness and anxiety being the most common emotions. However, individuals also documented a significant number of positive events, particularly in the categories ‘birth, health’ and ‘partnership, disease’.

Our findings expand on the conventional concept of disease progression in MS, which is structured around ‘medical milestones’, including diagnosis, treatment, relapses, or symptom changes (e.g., measured by the widely established ‘Expanded Disability Status Scale’). We found that most documented events were directly disease-related (e.g., diagnosis, symptoms, medication). However, our findings also highlight the importance of MS-related life events such as key human life goals (e.g., work, family, pregnancy), which tend to be negatively affected by MS. This is noteworthy as participants were free to choose and prioritize which MS-related life events they considered the most important and wished to share. The relevance of such ‘non-medical milestones’ is consistent with previous research and our own experience at the SMSR where persons with MS often referred to direct and indirect effects of their MS on work as well as social and family life. The confirmation of this experience-based prediction is thus one of several key findings of the present study.

Participants were invited to document the most significant events in their lives with MS, starting from the time of diagnosis to the present day. The majority of participants actually began their story with diagnosis, making the topic category ‘diagnosis’ highly prevalent. In terms of less prevalent topic categories, it is important to consider that the occurrence and/or timing of certain events (e.g., the need for a wheelchair) also depends on the duration of the MS and the nature of the disease progress (e.g., relapsing-remitting MS compared to primary progressive MS). Most event descriptions were associated with negative emotions (particularly anxiety and sadness), which is not surprising given the increasing burden faced by persons with MS as the disease progresses. For example, the topic category "work" often concerned degenerating skills and/or loss of opportunities (e.g., job loss). It is important to mention that our findings from language model-based emotion analysis aligned well with the survey-based emotion ratings for the events where participants had provided it. This attests to the validity of our emotion analysis. Our results complement previous qualitative research findings which indicate that being diagnosed with MS often represents a turning point in the individual’s life associated with denial, loss of confidence, as well as concealment [26].

It is also important to mention that in the present study a number of event descriptions were classified as predominantly positive (particularly feelings of joy). This is particularly the case for the topic ‘birth, health’. Typical negative events concerned, for instance, miscarriage or break-up with a partner. Positive events often pertained to giving birth and/or having a complication-free pregnancy or finding a new partner. The relevance and heterogeneity of these topics is not surprising–starting a family and parenthood are linked to common human life goals. However, achieving these goals is often complicated for persons with MS who may need to overcome more hurdles than healthy individuals (e.g., dropping or reducing medication before pregnancy). Also, the category ‘rehabilitation/wheelchair’ had noticeably low values for sadness and anxiety. This is due to the fact that this category of topics consists of very heterogeneous content. Rehabilitation was most often perceived as positive, while the need for a wheelchair or the prospect of needing one was negative. In other topic categories, similar positive event classifications occurred. Despite the physical and mental burden associated with MS, previous research has emphasized the importance of individuals’ psychosocial adaptation to their new situation. For example, studies have found that persons with MS experience stronger personal connections with others following their diagnosis, and that they generally reassess what is important to them in life [27,28]. Although the disability-related adjustments in work and personal life are initially experienced as very painful, many persons with MS later report a general improvement in their quality of life when they regain freedom or mobility. Reconciliation of one’s identity, strengthening personal relationships, and reprioritizing life issues are discussed as important factors in this psychosocial adjustment process [27,28].

### Limitations and Future Research

The present research has several limitations which merit consideration. First, the event descriptions differ substantially in their level of detail. While a number of participants provided mainly keywords, some provided comprehensive descriptions detailing thoughts and emotional experiences. Very short text entries lack context information and are therefore prone to misinterpretation. In terms of the overarching topics, we considered both short and detailed texts as informative sources of information as our analysis was based on nouns only, which were almost always present in both short and long text descriptions. Given the novelty of our methods and the specific nature of the data (MS context), we incorporated several manual steps to ensure the integrity of the methodology–a somewhat time-consuming process. Future research would therefore benefit from studies that evaluate the performance of such open-source language models on various health-related datasets (e.g., those that contain numerous medical terms and those that use everyday language). Second, there may have been self-selection in recruitment in the sense that mainly persons with MS who had a desire to tell their story participated in the study. In general, persons with a recent MS diagnosis, a milder MS disease course, or a very advanced disease stage (e.g. those who are entirely dependent on external help) are somewhat underrepresented in the SMSR. [47] Finally, our analytic approach is suitable for revealing general patterns in the text. A more fine-grained investigation of individual-level patterns and their contextual meaning would still require a more detailed review of the text entries in combination with additional individual-level context data (such as information on MS type and progression, wellbeing measures).

A current controversial debate concerns the limitations and risks of using artificial intelligence, which NLP is a subdiscipline of, in the exploration of health data. At its core, this discussion revolves around the risk of potential biases that the model has learned through the training data. In the present research, we have implemented pre-trained language models for automatic text translation and emotion classification. Instead, the LDA topic modelling technique is not subject to a priori bias as it does not rely on a pre-trained language model. With respect to automated text translations, a potential bias in our study is unlikely because all translations were manually reviewed by native-level speakers of the respective languages who also had expertise in MS. The pre-trained language model we implemented for emotion classification was fine-tuned on several public datasets which mainly include social media posts which that have been labelled for emotion categories by manual annotators [41]. To ensure that emotions would correctly classify the data in our study, we pre-tested several models as described in the methods section. The emotion classifications were also manually reviewed by CH, which verified that the overall quality of the classifications was high and appropriate. However, future research would benefit from emotion classification models trained on diary or patient narrative data to ensure high model performance.

Our findings have several implications for future research into the life stories of persons with MS. Future research on complex health conditions may benefit from a citizen science approach. Leveraging the mostly untapped expertise of ‘citizen scientists’ for research purposes may help uncover blind spots in health research. Their knowledge may also help pave the way for tailoring better disease management strategies (including medical, symptomatic, and lifestyle aspects) to individual needs, contexts, and everyday challenges. Language represents a key medium for harnessing the expertise of citizen scientists. Future research would also benefit from further development of language models that have been trained on data from individuals with a particular health condition and are therefore more accurate when applied to new data in a similar context. However, persons with severe chronic conditions may struggle to write longer text and default to using abbreviation and providing keywords instead of a coherent text, ultimately limiting the automatic text analytics. A promising avenue for future research would thus be daily-life speech sampling. The emergence of open-source speech-to-text models might circumvent the need to rely on commercial providers and pave the way for revolutionizing daily-life research. Finally, the high participation of registry participants in project development and participation indicates that many individuals with MS have experiences that they are willing to share. Our experience is indeed that the SMSR participants’ motivation for participating in research is often rooted in their wish to contribute to advancement which will benefit others in the long term. Future research may benefit from intensifying citizen-science collaborations to deepen our understanding of how MS impacts on individuals’ everyday experience and what is most needed to improve the quality of life for persons with MS.

## Conclusion

The present study investigated which categories of life events persons with multiple sclerosis (MS) perceive as central to their MS by combining citizens. This group of individuals is highly heterogeneous in terms of their life experiences, as they are often diagnosed at a younger age—some at 20, others at 35—and their life situations and experiences can be very different. Our work demonstrates how NLP and ML techniques can conveniently uncover previously underappreciated non-medical life experiences in a large sample of persons with MS.

We found that important life events for persons with MS go beyond typical clinical milestones such as diagnosis and changes in symptoms or treatment. Individuals’ lives can be fundamentally changed by the disease, affecting important areas of life such as interpersonal relationships, the ability to work or start a family. Our research highlights the many ways in which the lives of people with MS can be affected, all of which can mean painful loss—of relationships, abilities, or social status. Both in the detail of our work and in the scope of our participants, we want to raise awareness among practitioners so that they keep this in mind when they see their patients. As such, our findings are also useful in informing more comprehensive support and management approaches for persons with MS.

## Supporting information

S1 Text How is citizen science being implemented by the SMSR? (DOCX)

S1 Fig The ‘My life with MS’-survey. (DOCX)

S2 Fig Histogram depicting the frequency of the different numbers of words per text. (DOCX)

S3 Fig Coherence score for an increasing number of topics. (DOCX)

S4 Fig Emotion probability scores for ‘sadness’, ‘fear’, ‘anger’, ‘joy’, ‘surprise’, and ‘disgust’ are displayed separately for the eight topic categories. (DOCX)

S5 Fig Correlation plot displaying the relationship between the emotion scores obtained from the language model-based emotion analysis and the binary survey ratings via point-biserial correlation coefficients. (DOCX)

S6 Fig Bar chart showing the frequency of topic categories (present vs. not present) for all study participants. (DOCX)
