# Supplementary material for: Blending citizen science with natural language processing and machine learning: Understanding the experience of living with multiple sclerosis
Source: PLOS Digit Health. 2023 Aug 2;2(8):e0000305. doi: 10.1371/journal.pdig.0000305 (PMC10395829; doi:10.1371/journal.pdig.0000305)
Supplement: S1 Text — (DOCX) [file pdig.0000305.s001.docx]

**S1 Text. How is citizen science being implemented by the SMSR?**

Participants can get involved in SMSR research in a variety of ways. The majority of participants contribute data by completing regular surveys, but a number of participants also co-create research. This is possible because the SMSR has people with MS in all of its decision-making bodies, such as the Medical-Scientific Advisory Board and the Research Committee. Citizen scientists in this role participate in fundamental decisions about research priorities and suggest under-researched topics of high relevance to people with MS.

To provide feedback to participants and discuss research with them interactively, SMSR conducts regular webinars on selected topics. These webinars are open to all SMSR participants, people with MS in general and other interested members of the public. The webinars are designed both to keep SMSR participants informed of research findings and to provide an opportunity to participate in the research through discussion. In addition to the webinars, there are several other information channels through which the SMSR provides feedback to its participants: Research articles and lay summaries in the official Swiss languages of German, French and Italian are made available online by the SMSR's sponsor, the Swiss MS Society. The SMSR also regularly publishes short lay articles summarizing ongoing research, which are also made available online by the Swiss MS Society. Finally, the SMSR provides all participants with an annual report summarizing the year's research.

Participants do not receive additional incentives other than being kept informed and involved in ongoing research and MS-related topics in general. Our experience at the SMSR is that many participants are highly intrinsically motivated to participate and wish to make a contribution that will benefit future persons with MS. We have received this feedback very often, both in personal conversations, in messages and as free text responses in our surveys. This intrinsic motivation and desire to contribute also suggests that the overall quality of the data we receive is high.
