# Supplementary figures and images for: Blending citizen science with natural language processing and machine learning: Understanding the experience of living with multiple sclerosis
Source: PLOS Digit Health. 2023 Aug 2;2(8):e0000305. doi: 10.1371/journal.pdig.0000305 (PMC10395829; doi:10.1371/journal.pdig.0000305)

**S1 Fig. The ‘My life with MS’-survey**


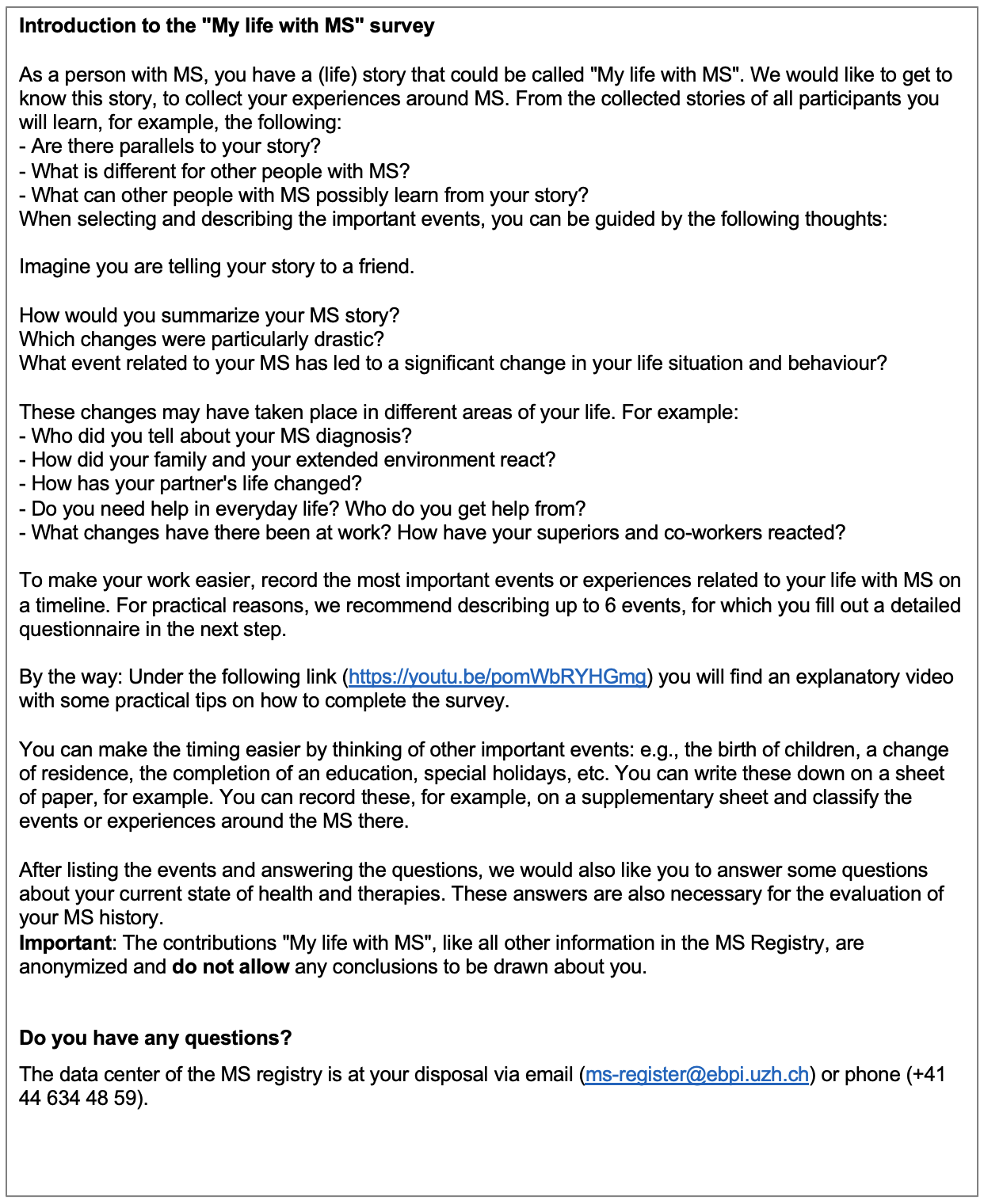


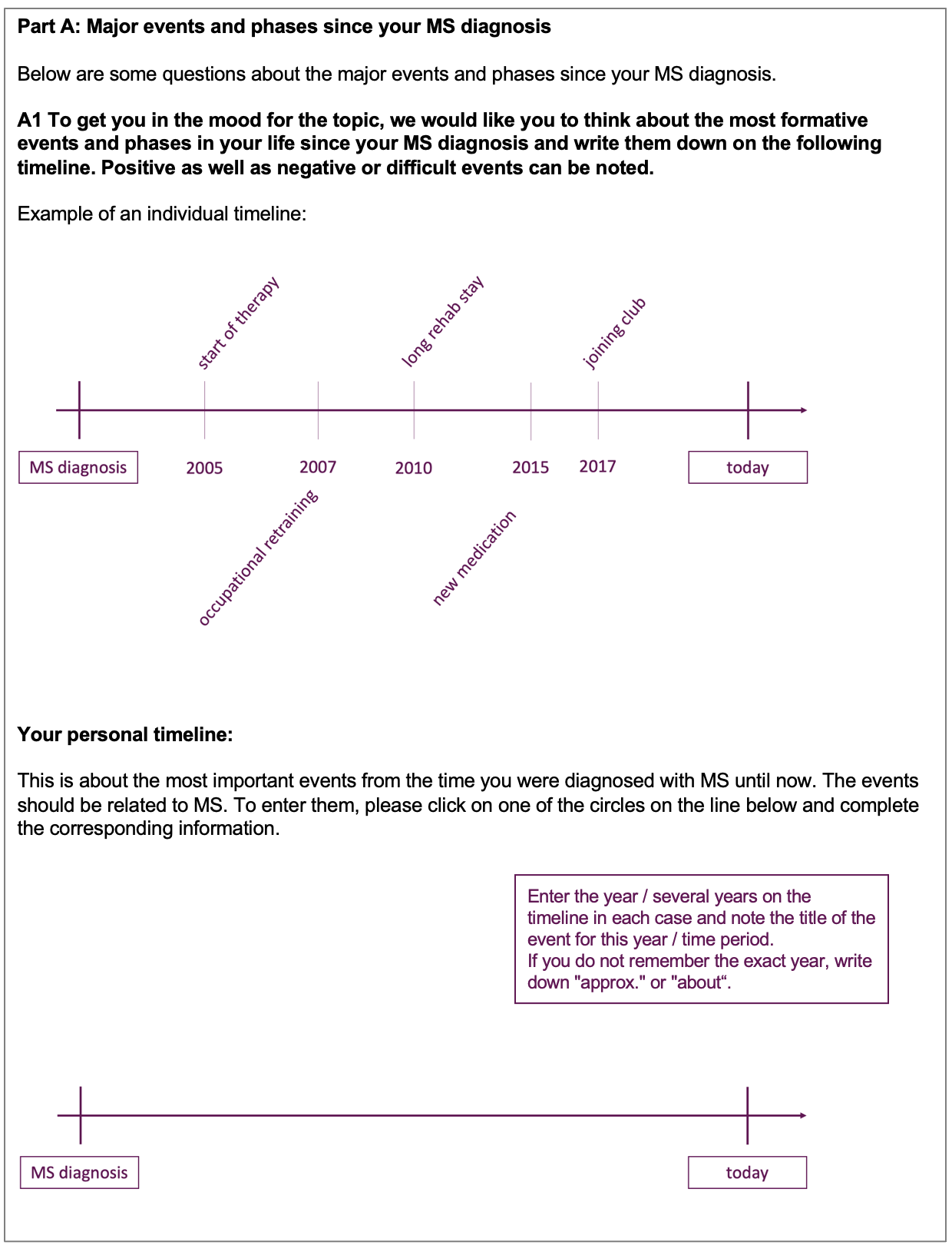


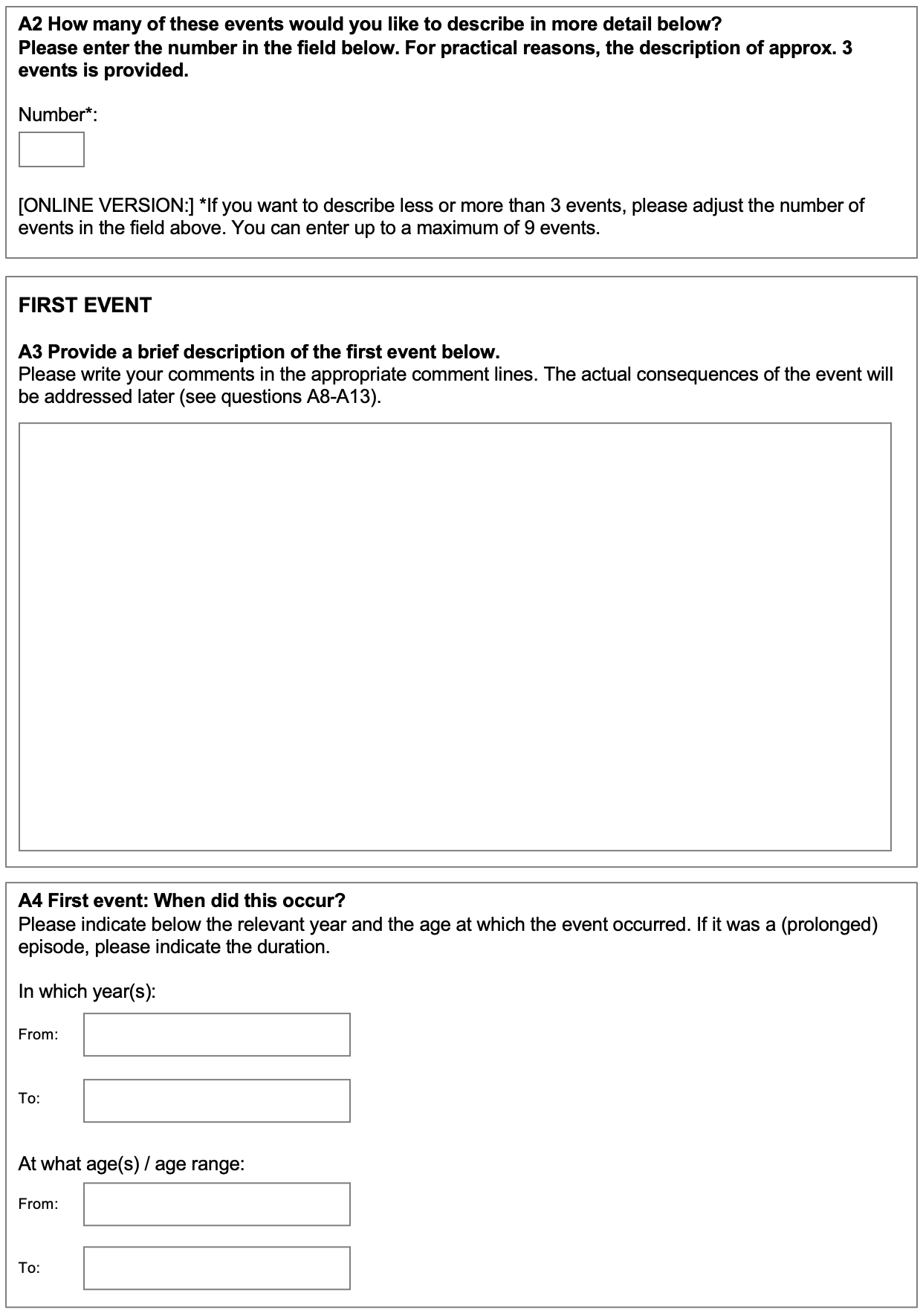


**
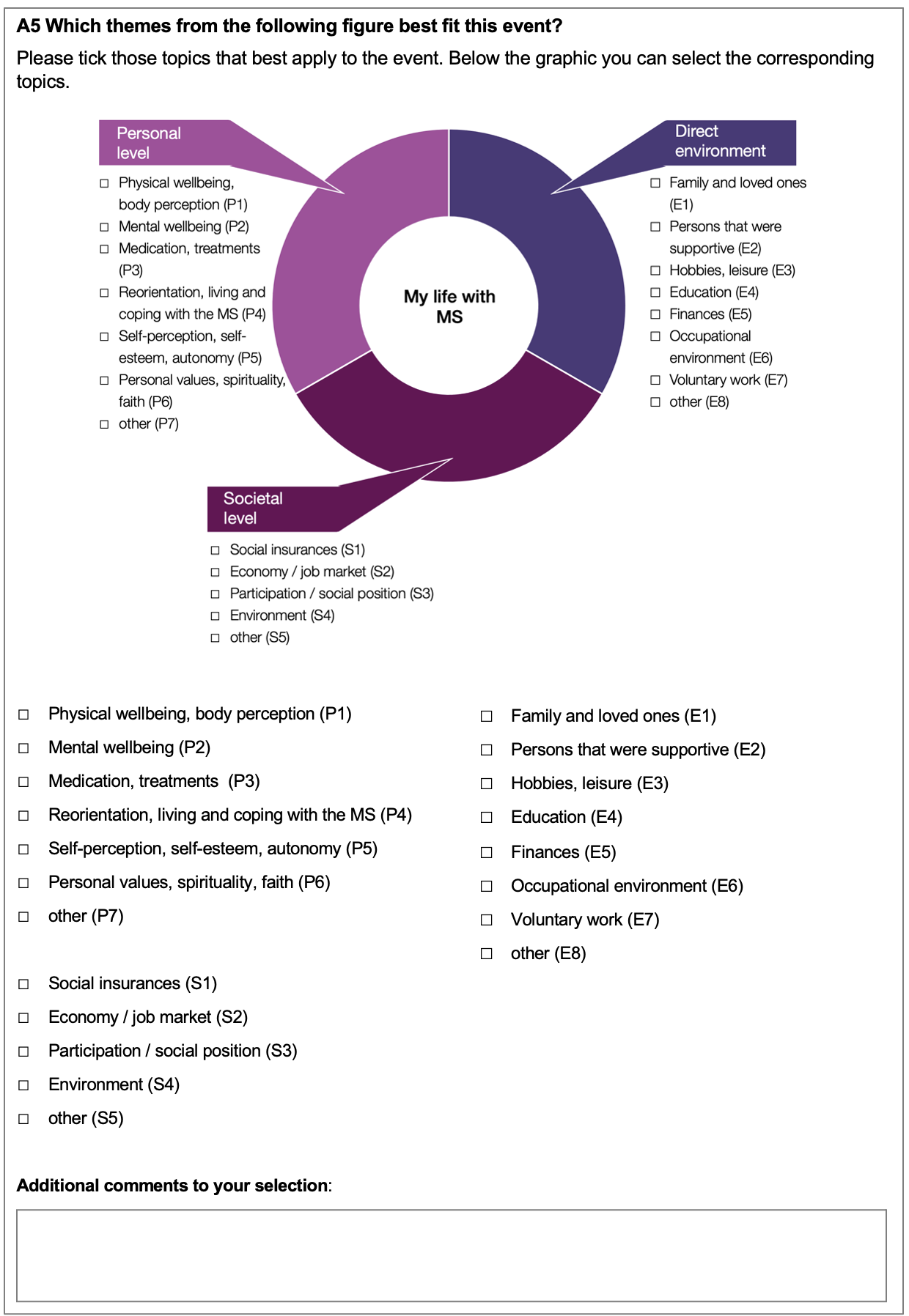
**


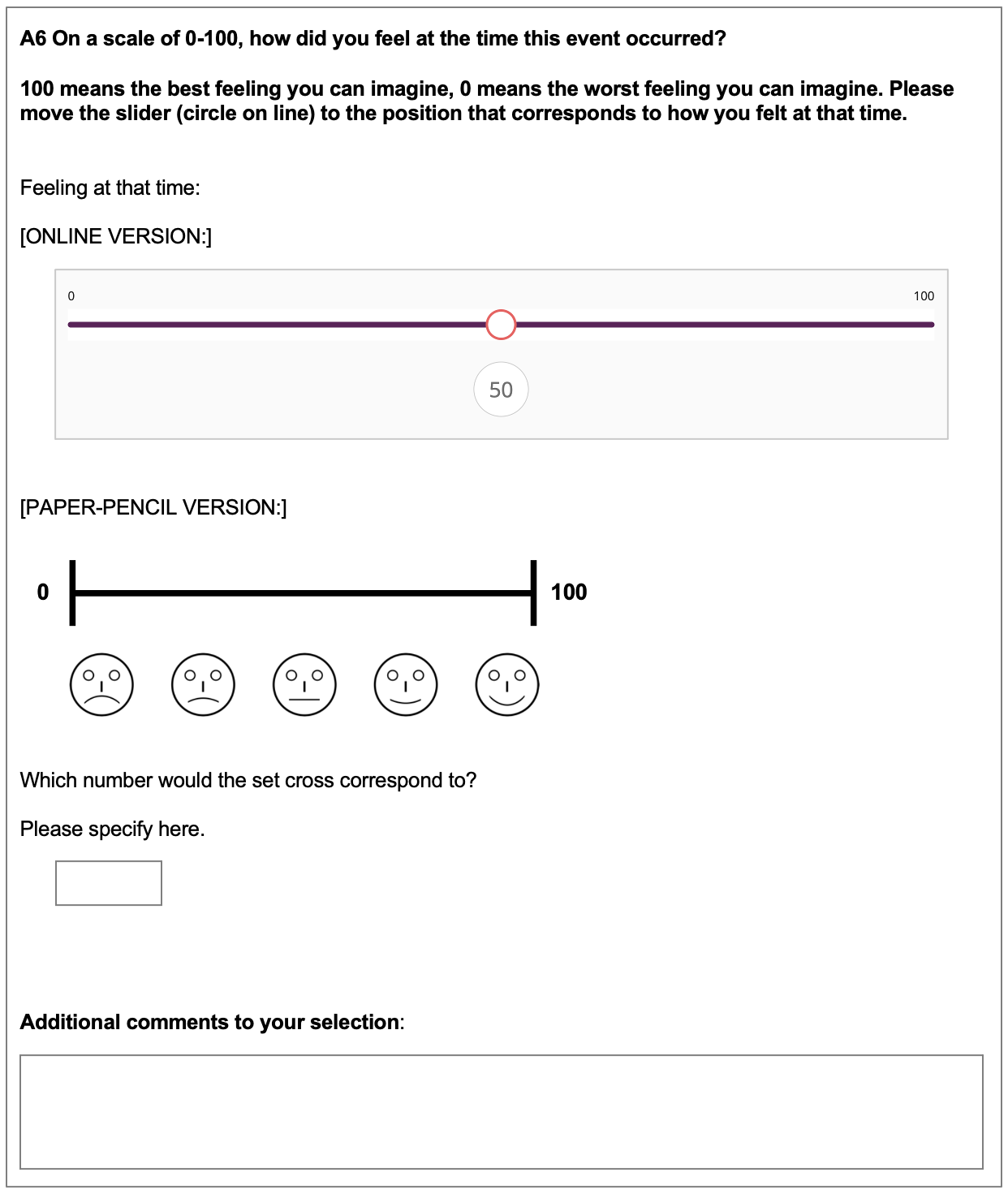


**
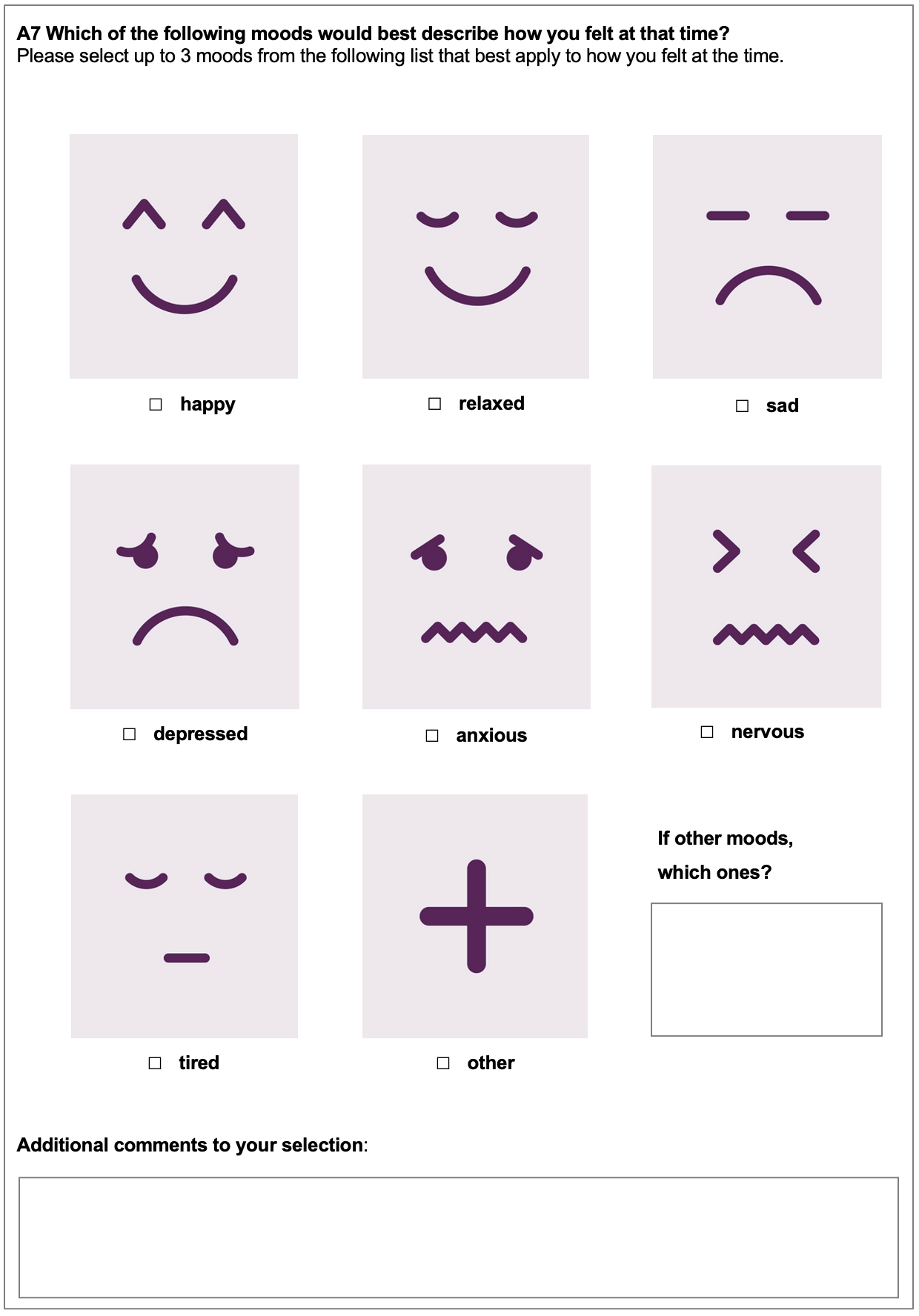
**


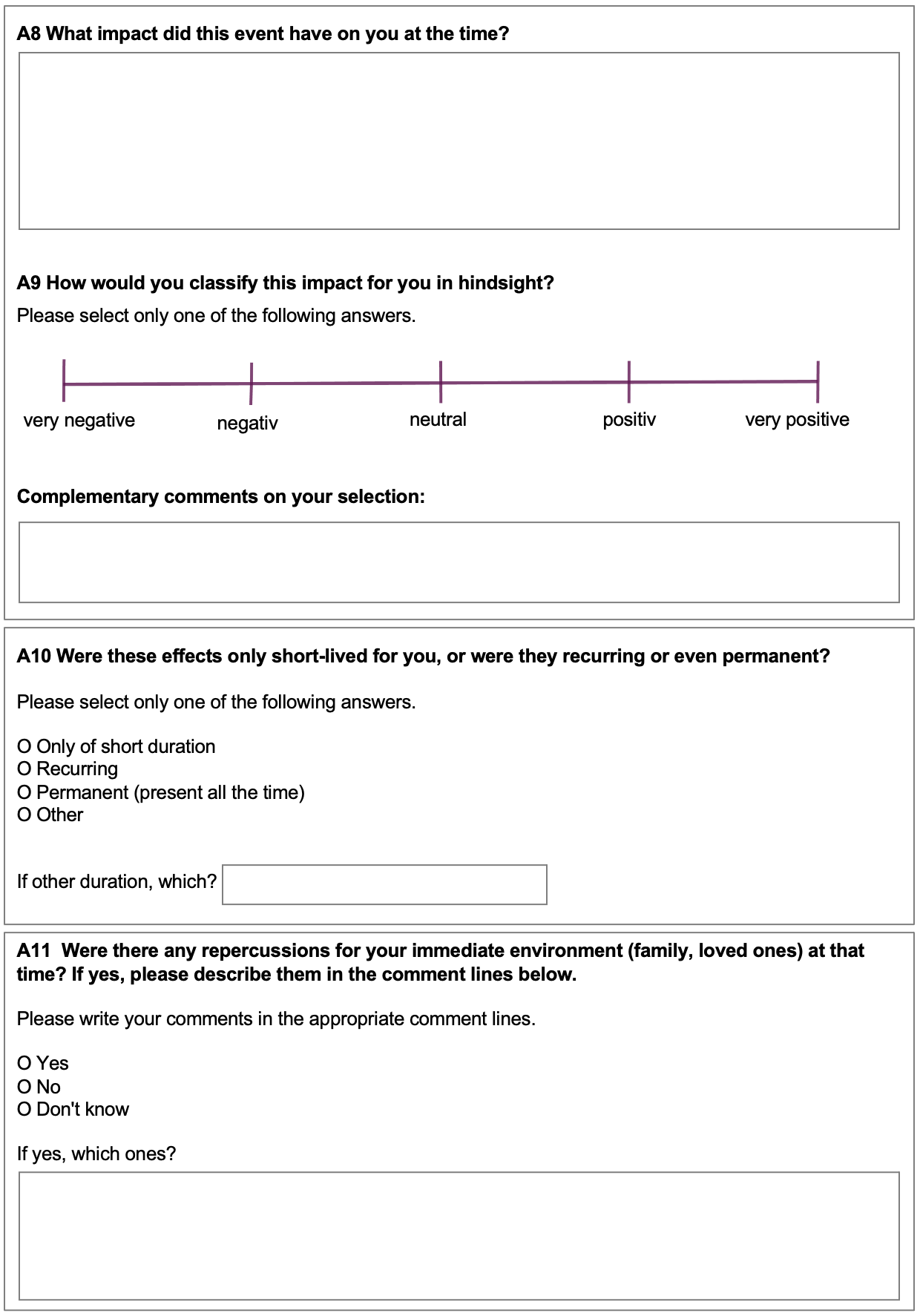


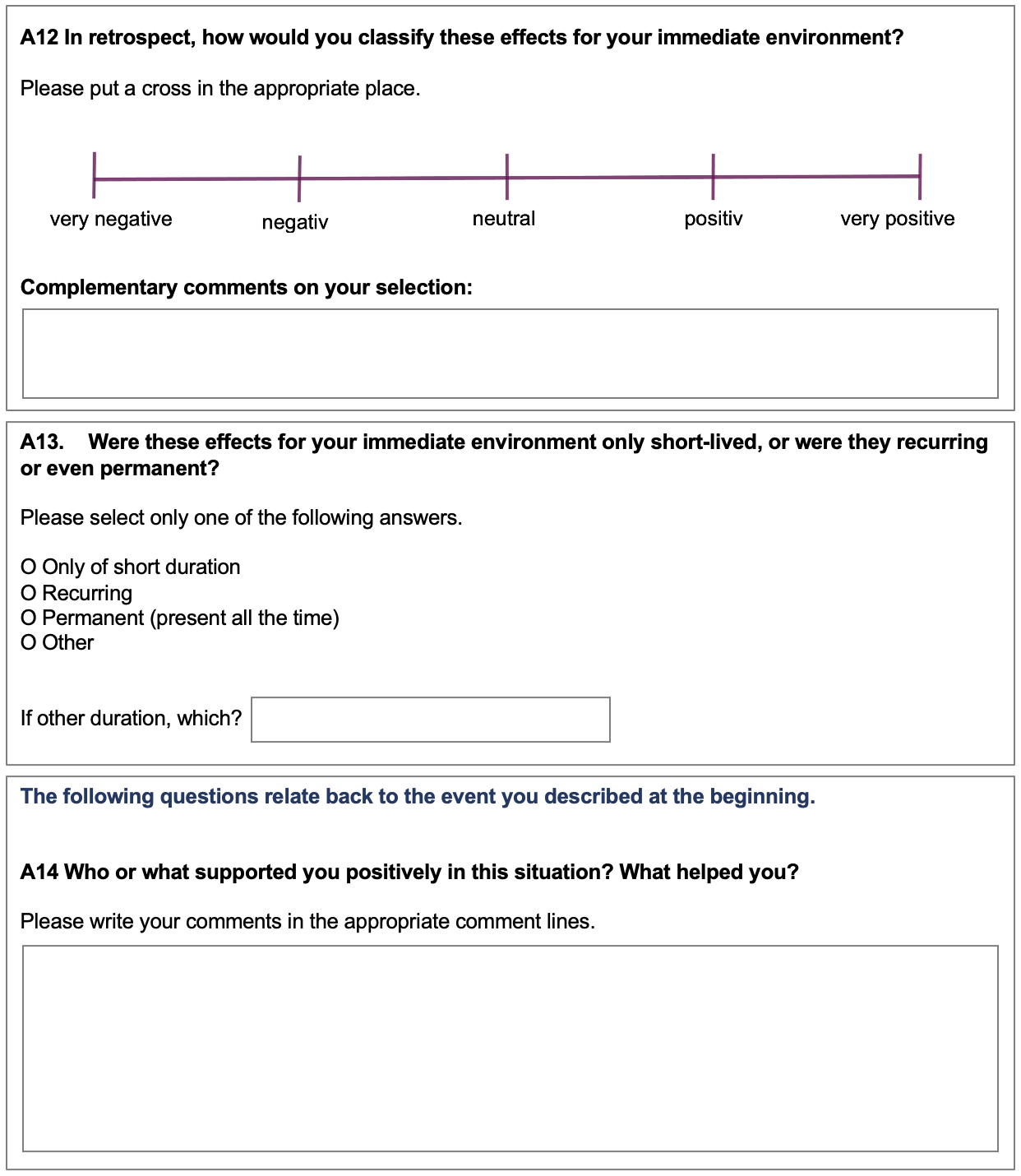


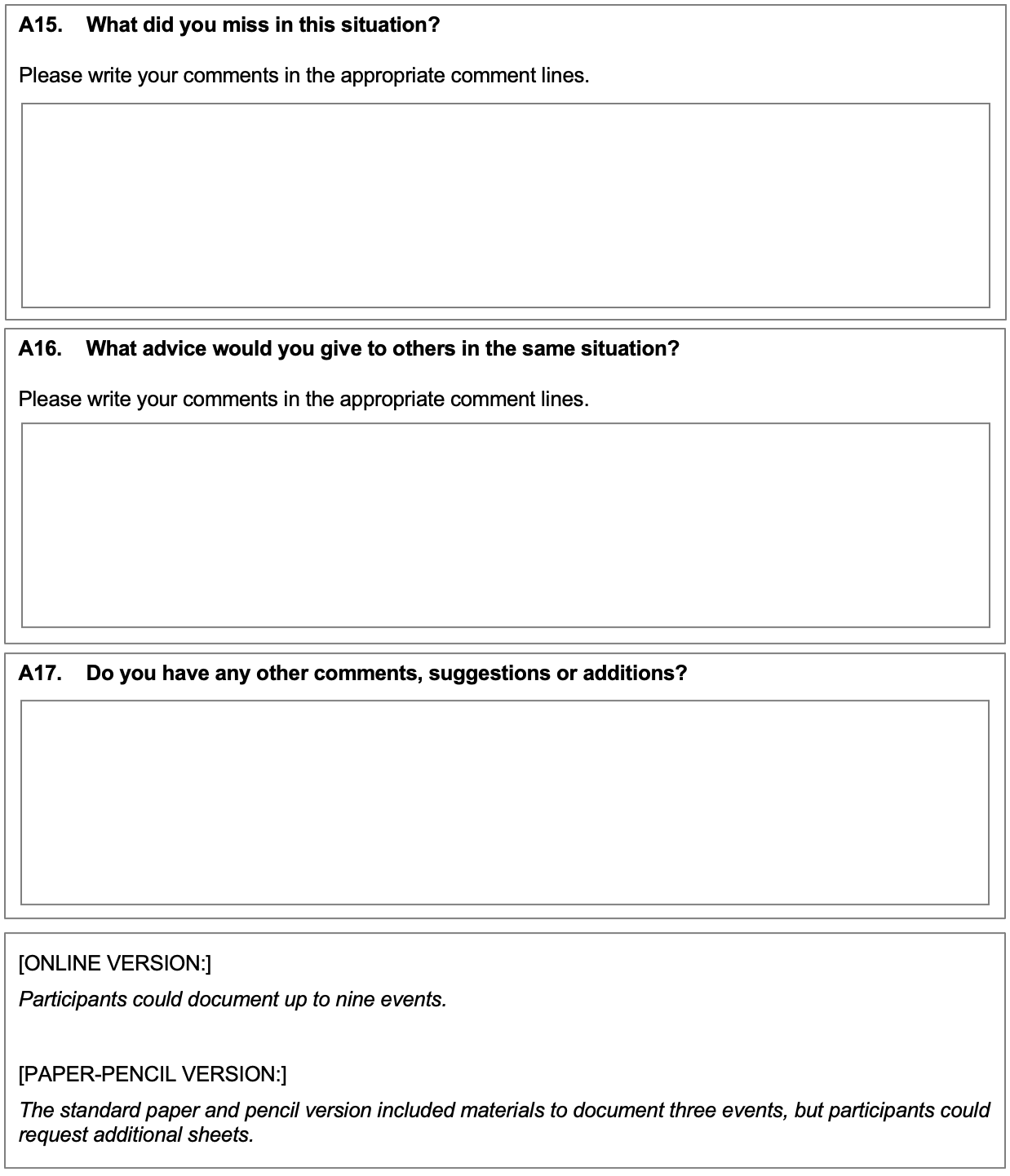


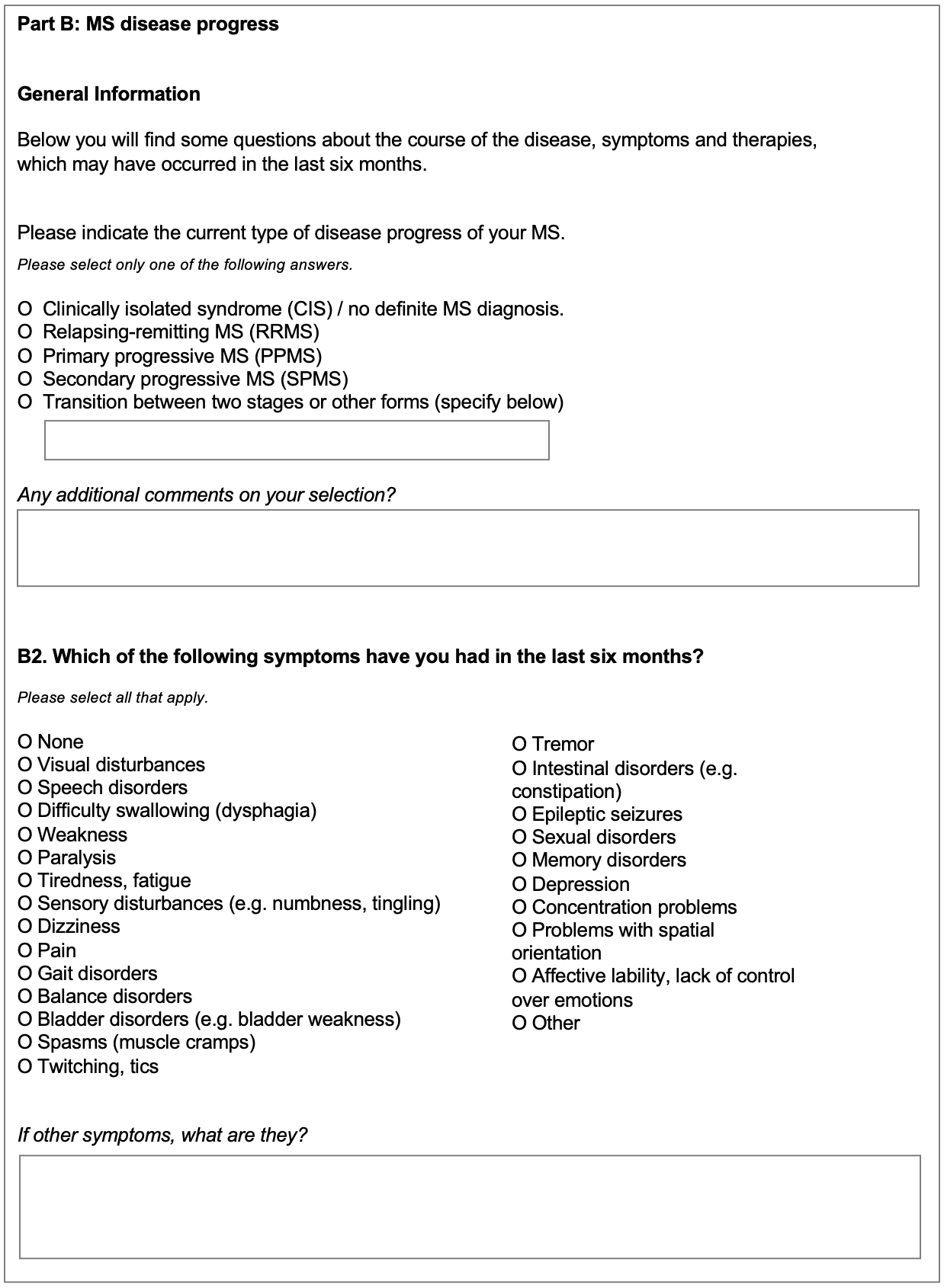


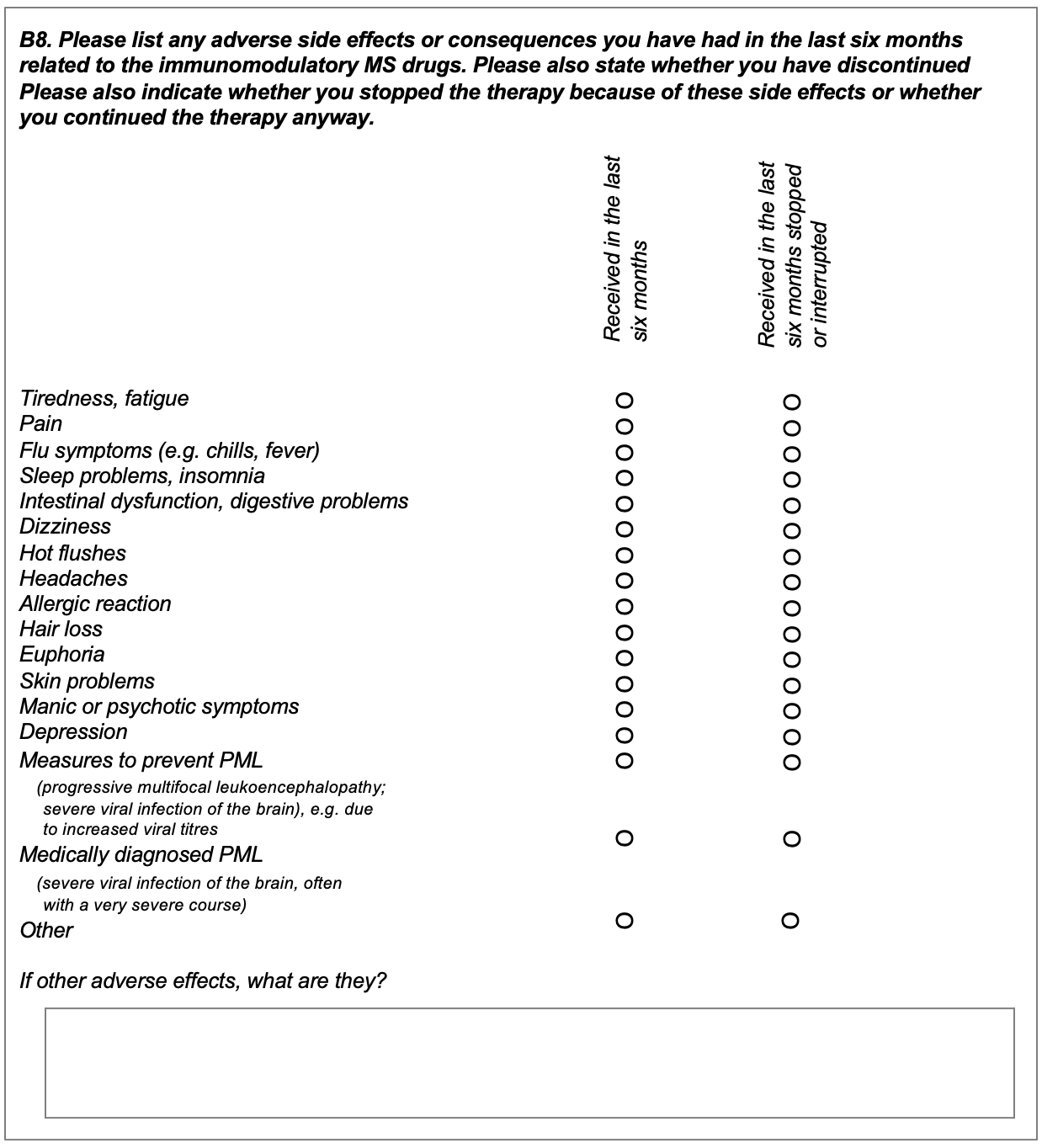


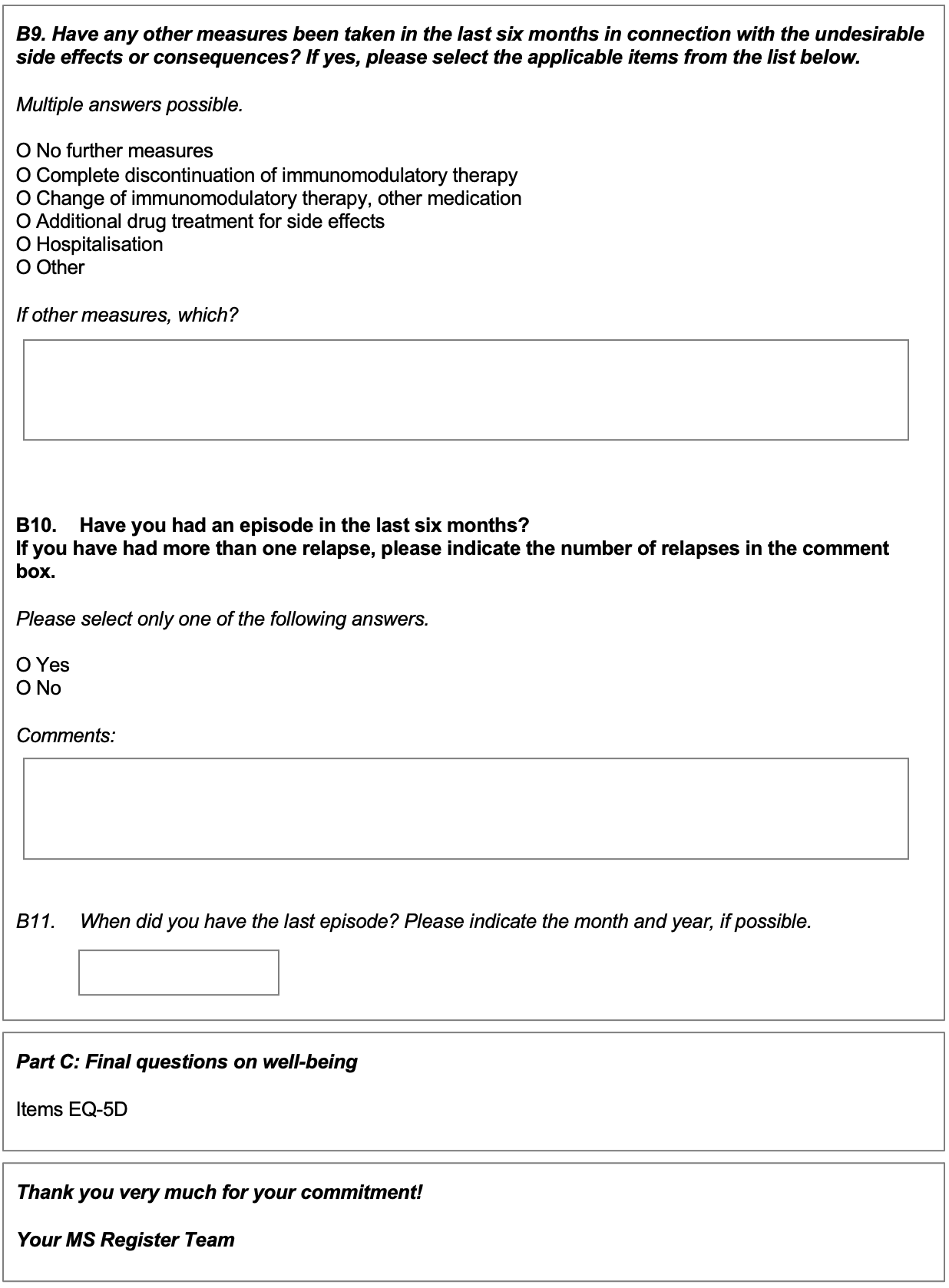

Supplement: S1 Fig — (DOCX) [file pdig.0000305.s002.docx]
