# Supplementary material for: Blending citizen science with natural language processing and machine learning: Understanding the experience of living with multiple sclerosis
Source: PLOS Digit Health. 2023 Aug 2;2(8):e0000305. doi: 10.1371/journal.pdig.0000305 (PMC10395829; doi:10.1371/journal.pdig.0000305)
Supplement: S2 Fig — (DOCX) [file pdig.0000305.s003.docx]

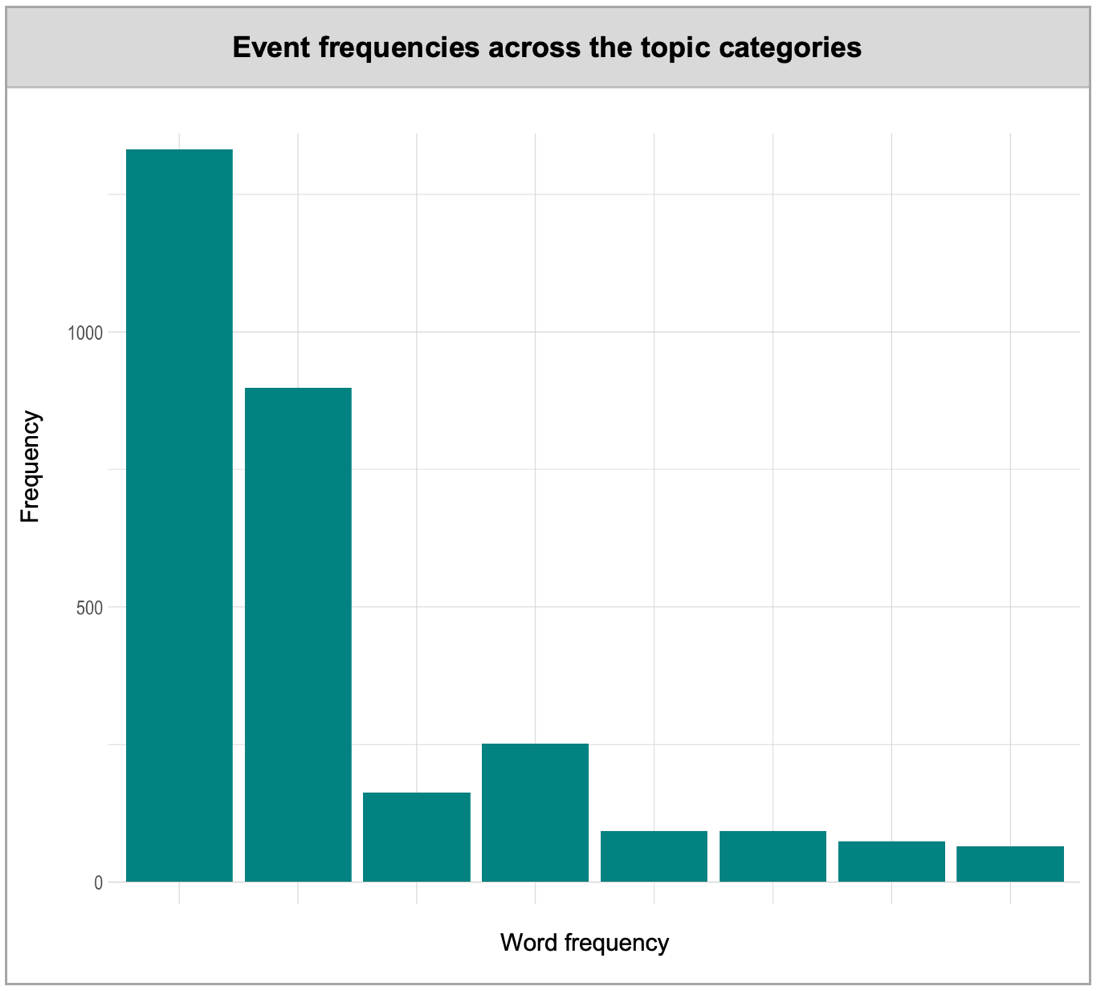


**S2 Fig**. Histogram depicting the frequency of the different numbers of words per text. The number of words per entry is plotted along the x-axis. Word frequency is plotted against the y-axis.
