# Supplementary material for: Blending citizen science with natural language processing and machine learning: Understanding the experience of living with multiple sclerosis
Source: PLOS Digit Health. 2023 Aug 2;2(8):e0000305. doi: 10.1371/journal.pdig.0000305 (PMC10395829; doi:10.1371/journal.pdig.0000305)
Supplement: S3 Fig — (DOCX) [file pdig.0000305.s004.docx]

**
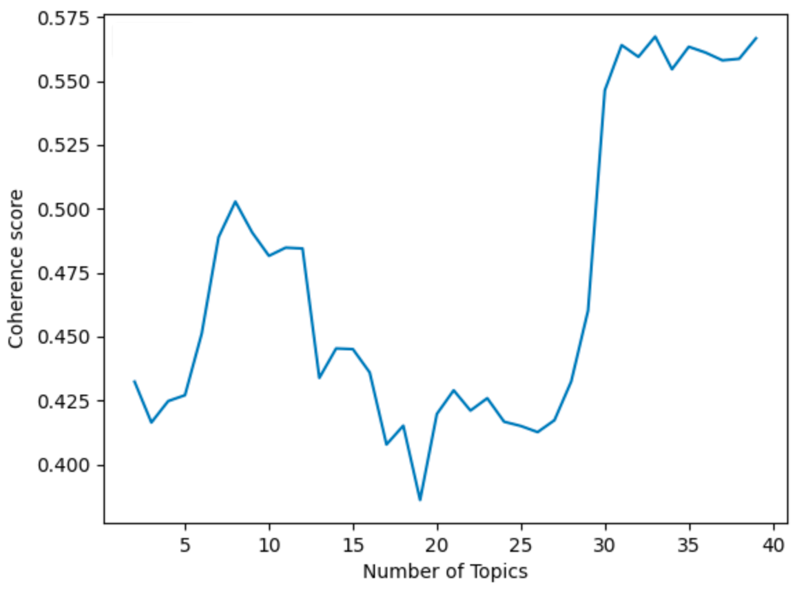
**

**S3 Fig**. Coherence score for an increasing number of topics. The graph depicts that the topic models with 8 and 30 or more topics results the highest topic coherence (coherence score: 0.50 and >0.50, respectively). The number of modelled topics is plotted along the x-axis. Coherence scores are plotted along the y-axis. Topic coherence refers to the semantic similarity of words allocated to a distinct topic and constitutes a key goodness of fit measure for topic models. The full possible range of coherence scores is between 0 (no topic coherence) and 1 (complete topic coherence). An 8-topic model provides the optimal modelling solution for the data as indicated by the highest coherence score.
