# Supplementary material for: Blending citizen science with natural language processing and machine learning: Understanding the experience of living with multiple sclerosis
Source: PLOS Digit Health. 2023 Aug 2;2(8):e0000305. doi: 10.1371/journal.pdig.0000305 (PMC10395829; doi:10.1371/journal.pdig.0000305)
Supplement: S4 Fig — (DOCX) [file pdig.0000305.s005.docx]

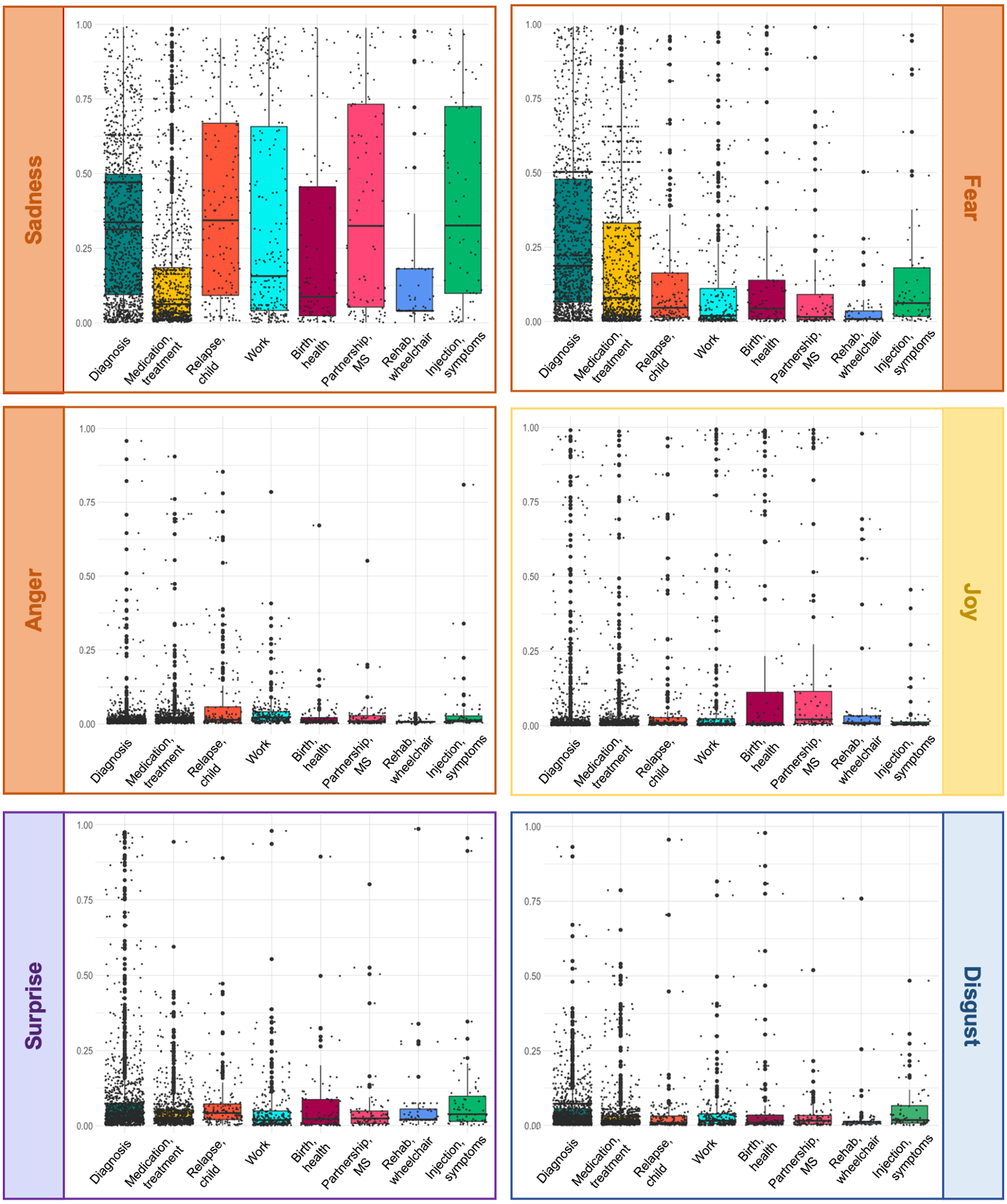


**S4 Fig**. Emotion probability scores for ‘sadness’, ‘fear’, ‘anger’, ‘joy’, ‘surprise’, and ‘disgust’ are displayed separately for the eight topic categories. Only events for which (1) the respective category is the "main category" and (2) the assignment to this main category is at least one third (33%) are included in the graph. Emotion probability scores range from 0 to 1. Higher scores indicate a higher probability for a text description being corresponding to the respective emotion category. Emotion probability scores are displayed using boxplots. The boxplot’s middle line refers to a topic category’s median, the box itself displays the interquartile range (IQR; the range from the 25^th^ to the 75^th^ percentile), and the whiskers mark the minimum (25^th^−1.5*IQR) and maximum (75^th^−1.5*IQR). The thick dots represent outliers beyond the minimum/maximum. The small dots display the raw data.
