# Supplementary material for: Blending citizen science with natural language processing and machine learning: Understanding the experience of living with multiple sclerosis
Source: PLOS Digit Health. 2023 Aug 2;2(8):e0000305. doi: 10.1371/journal.pdig.0000305 (PMC10395829; doi:10.1371/journal.pdig.0000305)
Supplement: S5 Fig — (DOCX) [file pdig.0000305.s006.docx]

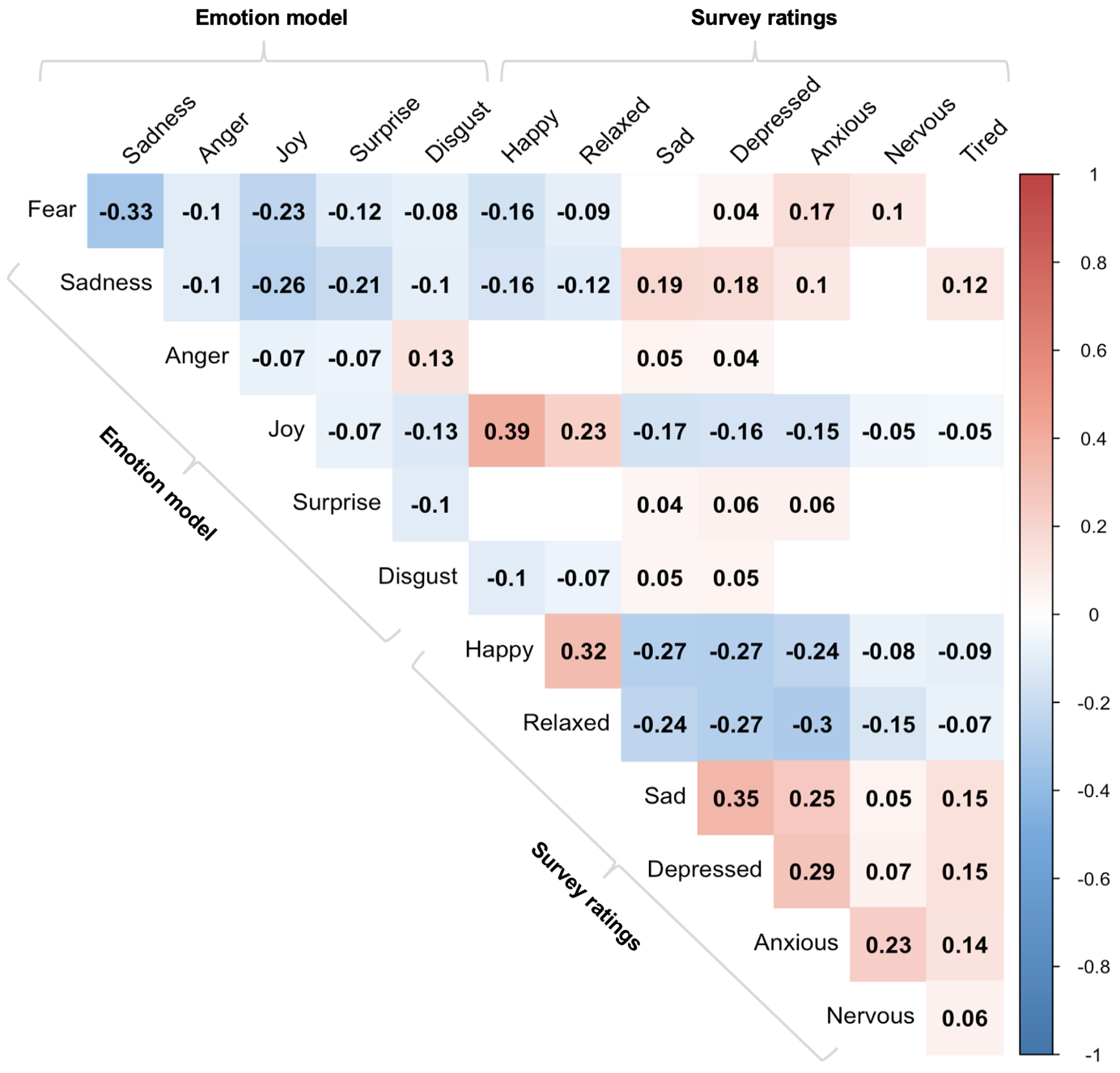


**S5 Fig**. Correlation plot displaying the relationship between the emotion scores obtained from the language model-based emotion analysis (probability scores ranging between 0 and 1) and the binary survey ratings (emotion was present: 1; emotion was not present: 0) via point-biserial correlation coefficients. All correlation coefficients shown are also statistically significant (p < 0.05). Non-significant coefficients are not displayed. The background color of the coefficients visually indicates whether the correlation is positive (red) or negative (blue).
