# Supplementary material for: Blending citizen science with natural language processing and machine learning: Understanding the experience of living with multiple sclerosis
Source: PLOS Digit Health. 2023 Aug 2;2(8):e0000305. doi: 10.1371/journal.pdig.0000305 (PMC10395829; doi:10.1371/journal.pdig.0000305)
Supplement: S6 Fig — (DOCX) [file pdig.0000305.s007.docx]

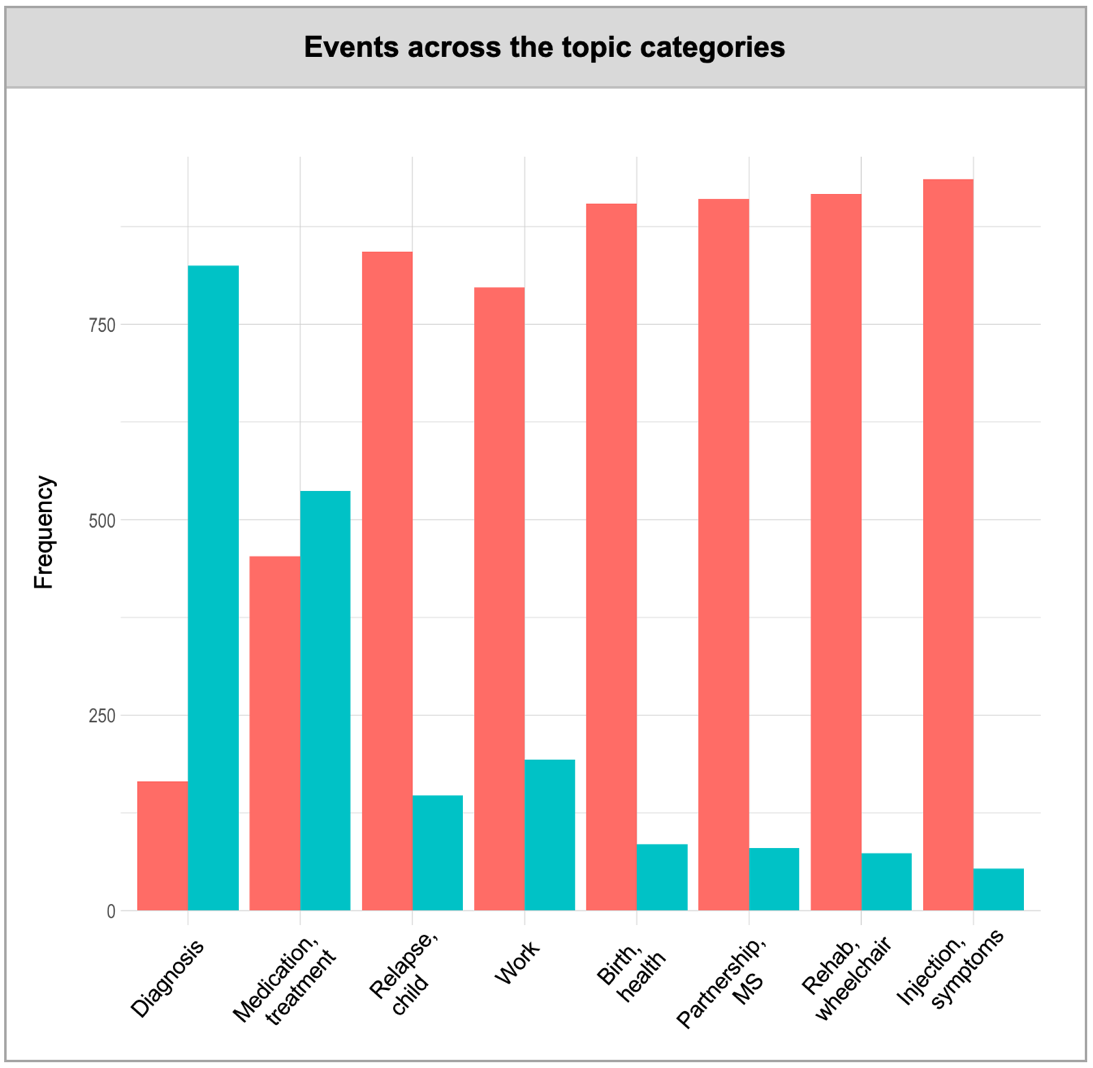


**S6 Fig**. Bar chart showing the frequency of topic categories (present vs. not present) for all study participants. The topic category is plotted along the x-axis. The frequency with which a topic is present (turquoise) or not present (red) in the overall sample is plotted along the y-axis.
